# Deficiency in catechol-o-methyltransferase is linked to a disruption of glucose homeostasis in mice

**DOI:** 10.1038/s41598-017-08513-w

**Published:** 2017-08-11

**Authors:** Megumi Kanasaki, Swayam Prakash Srivastava, Fan Yang, Ling Xu, Sumiyo Kudoh, Munehiro Kitada, Norikazu Ueki, Hyoh Kim, Jinpeng Li, Satoru Takeda, Keizo Kanasaki, Daisuke Koya

**Affiliations:** 1 Department of Diabetology & Endocrinology, Kanazawa Medical University, Ishikawa, Japan; 2 Department of Obstetrics and Gynecology, Juntendo University Faculty of Medicine, Tokyo, Japan; 3 Department of Medicine, Shiga University of Medical Science, Otsu, Shiga Japan; 4 Department of General Medicine, Kanazawa Medical University, Ishikawa, Japan; 5 Division of Anticipatory Molecular Food Science and Technology, Medical Research Institute, Kanazawa Medical University, Uchinada, Ishikawa 920-0293 Japan

## Abstract

2-methoxyestradiol (2-ME), an estrogen metabolite generated via catechol-o-methyltransferase (COMT), is multifunctional methoxy-catechol. Here, we report that COMT deficiency leads to glucose intolerance and 2-ME rescues COMT-deficient-associated metabolic defects. Liver COMT protein was suppressed in high fat diet (HFD)-fed or in pregnant mice. COMT suppression, by Ro41-0960 or siRNA, in HFD fed mice or in pregnant mice exacerbated glucose intolerance; 2-ME intervention ameliorated these defects. 2-ME effects on glucose tolerance were associated with AMPK phosphorylation in the liver and in islet cells. Metformin restored liver COMT protein levels, and metformin-induced liver AMPK phosphorylation was abolished by COMT inhibition. The amelioration in glucose tolerance by 2-ME was associated with biphasic insulin secretion in an environment-dependent manner. 2-ME-induced insulin secretion was associated with the AMPK phosphorylation, PDX-1 phosphorylation, and MST-1 suppression in MIN-6 cells. Furthermore 2-ME displayed PPARγ agonist-like activity. These results suggest that COMT is an enzyme to maintain glucose homeostasis and 2-ME is a potential endogenous multi-target anti-diabetic candidate.

## Introduction

Catechol-o-methyltransferase (COMT) is an enzyme responsible for the metabolism of catechols, such as catecholamines and catechol estrogens. Estradiol is catalyzed into hydroxyestradiol, one of the catechol estrogens, by cytochrome P450^1, 2^. Hydoxyestradiol is the substrate for the COMT, and COMT transmethylates hydroxyestradiol into 2-methoxyestradiol (2-ME)^1, 2^. Regarding the physiological role of 2-ME, deficiency in COMT and 2-ME leads to a preeclampsia-like phenotype in mice^3^ and have shown anti-inflammatory property both *in vivo* and *in vitro* ^4^. The human COMT gene exhibits functional SNPs, which can decrease protein stability and subsequent reduction in enzymatic activity (COMT^158Val-Met^)^5^. COMT^158Val-Met^ has been shown to associate with various psychiatric diseases^5^. Studies have suggested that COMT^158Val-Met^ may participate in obesity and diabetes^6–10^. A recent report envisaged, COMT rs4680 high-activity G-allele was found to associate with lower HbA1c level and modest protection from type 2 diabetes^10^.

Metabolic defects are also characteristic of preeclampsia^2, 11^, a COMT deficiency-associated disease. The cohort study recruited 3637 patients without gestational diabetes carrying singleton fetuses revealed that glucose intolerance in women without overt gestational diabetes was associated with the incidence of preeclampsia^12^. A prospective study showed that hyperinsulinemia at 20 weeks of gestation was associated with preeclampsia in African-Americans^13^. Glucose levels at 1 hour after a 50 g oral glucose tolerance test^14^ and insulin resistance^15, 16^ in early pregnancy was associated with the onset of preeclampsia. In contrast, women with a history of preeclampsia have been associated with a future metabolic risk for cardiovascular diseases including type 2 diabetes^17–19^. Such a risk of metabolic diseases in women with a preeclampsia history often has been explained by the hypothesis that vascular damage during pregnancy in preeclamptic women causes endothelial injuries that leads to metabolic defects associated cardiovascular diseases^17–19^; however, the possibility of either a shared genetic background or molecular mechanism has not been investigated.

Here, we hypothesized that COMT deficiency is a shared pathogenesis in the onset of the metabolic defects observed in the metabolic syndrome, type 2 diabetes, and preeclampsia.

## Results

### Suppression of COMT protein level in the liver of high fat diet fed mice, is associated with metabolic defects

**Figure 1:**
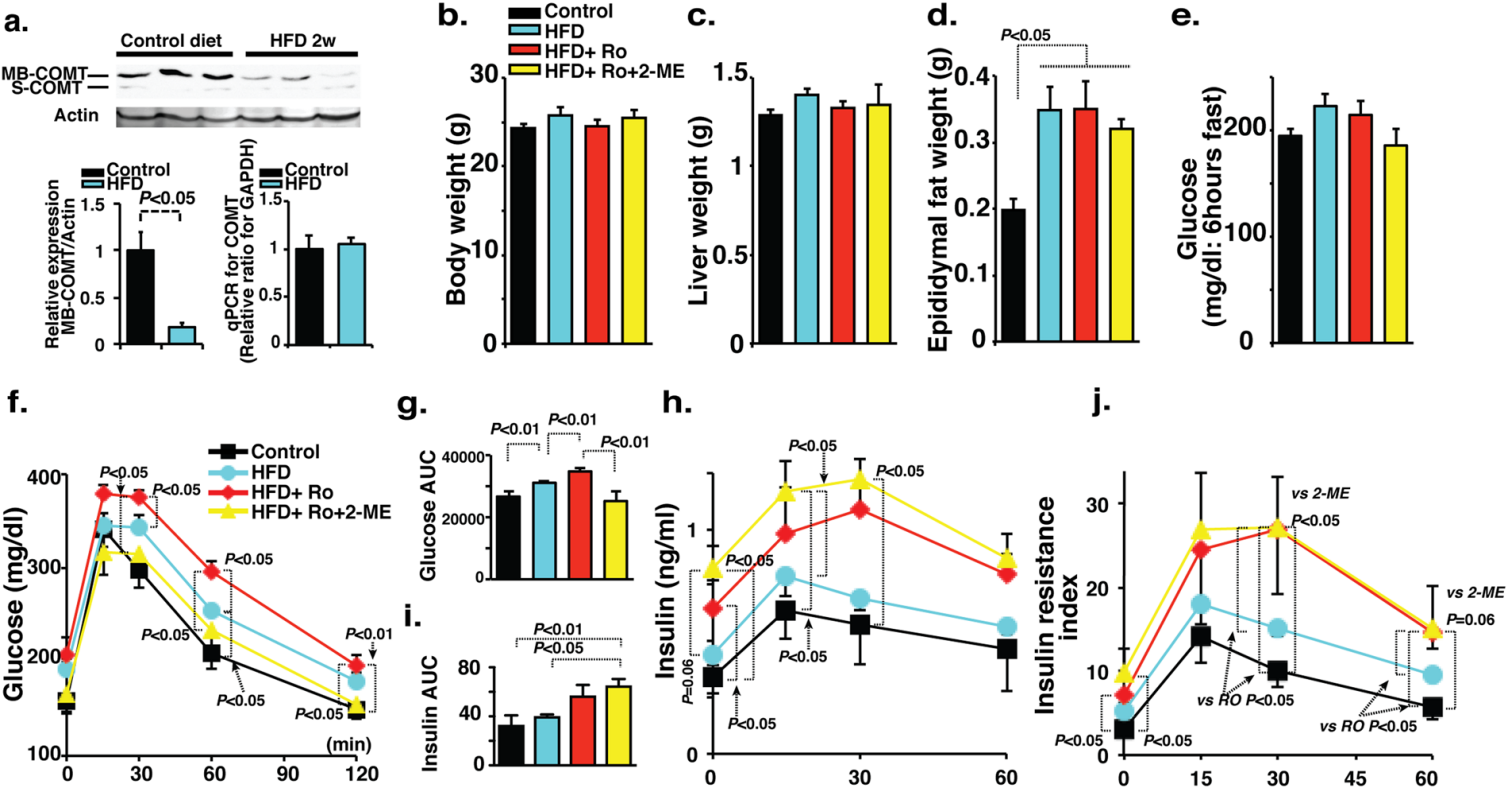
Inhibition of COMT exacerbates glucose tolerance defects in mice fed the HFD for 2 weeks. (**a**) Western blot analysis of COMT protein in the liver of HFD condition. The protein lysate (20 µg) was separated in polyacrylamide gels and transferred onto a PVDF membrane. The immunoreactive bands were analyzed using the ECL method. Representative blots from six independent experiments are shown. Cropped images were displayed and original blots are shown in the figure Supplementary 10. Quantification by densitometry is shown and reveals a significant reduction of the COMT protein level in mice fed an HFD for 2 weeks. N = 8 from each group were analyzed. Relative COMT gene expression by qPCR analysis in the liver of HFD mice expressing unaltered mRNA level. N = 6 from each group were analyzed. (**b**–**d**) The body, liver weight, and epididymal fat weights and fasting blood glucose levels were analyzed in control, HFD, COMT inhibitor (Ro41-0960)-treated HFD mice (HFD + Ro41-0960) and 2-ME intervened COMT inhibitor-treated HFD (HFD + Ro41-0960 + 2-ME) mice groups. N = 8 were analyzed in each mice group. (**e**) 6 hours fasting blood glucose levels were analyzed in control, HFD, COMT inhibitor (Ro41-0960)-treated HFD mice (HFD + Ro41-0960) and 2-ME intervened COMT inhibitor-treated HFD (HFD + Ro41-0960 + 2-ME) mice groups. N = 8 in each group. (**f**) IPGTT analysis. N = 8 were analyzed in each mice group. (**g**) The quantitative determination of glucose tolerance defects represented by the area under curve (AUC) value. (**h**) Insulin levels determined by ELISA. The insulin level was analyzed in triplicate by a sandwich ELISA method. N = 8 were analyzed in each mice group. (**i**) The AUC value of the insulin level. (**j**) Insulin resistance Index. The results are shown as the mean ± s.e.m. COMT inhibitor (Ro41-0960) was designated as Ro in the figure. N = 8 were analyzed in each mice group. Prism7.0 software was utilized for the statistical calculation. The Mann-Whitney test was carried out to determine of statistical significance.

The COMT protein is abundant in the liver when compared to other organs and the liver play essential roles in metabolic regulation. Therefore to determine whether COMT deficiency was associated with the metabolic syndrome, first we analyzed liver COMT protein levels in mice fed a high fat diet (HFD) for 2 weeks. Western blot analysis revealed a remarkable down regulation in COMT protein level in the liver of HFD mice compared with the liver of control mice (Fig. 1a). qPCR analysis revealed unaltered COMT mRNA expression levels in the livers of HFD mice (Fig. 1a), suggesting differences in COMT protein expression could be due to changes in translation, accumulation, break down, and location of the enzyme.

To further address the role of COMT suppression in the metabolic syndrome, we performed a COMT inhibitor (Ro41-0960) treatment in the HFD mice. After 2 weeks of the experimental protocol, body and liver weights were unchanged in all of the groups (Fig. 1b,c). The weight of epididymal fat was heavier in the HFD mice, and neither the COMT-inhibitor nor 2-ME altered the weight significantly (Fig. 1d). The mice of the entire group after a 6-h fast did not display significant differences in their blood glucose levels (Fig. 1e). Intra-peritoneal glucose tolerance test (IPGTT) revealed impaired glucose tolerance after 2 weeks of HFD feeding (Fig. 1f,g). Treatment with the COMT inhibitor for the last 1 week exacerbated glucose intolerance in HFD mice (Fig. 1f,g). 2-ME injection in COMT-inhibitor-treated HFD mice restored glucose tolerance (Fig. 1f,g). Similar trends of COMT inhibitor and 2-ME on glucose tolerance were observed in normal diet fed mice (Fig. S1a,b). COMT inhibitor treatment in HFD mice resulted in an elevated trend of insulin levels when compared to that of control mice (Fig. 1h,i). COMT inhibitor treatment increased HFD-mediated augmentation of insulin levels, even though blood glucose levels appeared higher (Fig. 1h,i). Interestingly, 2-ME intervention in COMT inhibitor-treated HFD mice caused elevation in the circulatory insulin levels along with the normalization of blood glucose levels (Fig. 1f–i), suggesting that 2-ME could ameliorate glucose stimulated insulin secretion in COMT inhibitor-treated HFD mice; 2-ME effects on insulin levels were different in normal diet fed COMT inhibitor treated mice (Fig. S1c,d). Using a previously reported formula^20^, the insulin resistance estimation revealed that HFD feeding for 2 weeks induced an enhancement in the trend of insulin resistance, and such HFD-enhanced insulin resistance was further augmented by COMT inhibitor administration (Fig. 1j). 2-ME did not ameliorate insulin resistance index in this experimental set (Fig. 1j).

### COMT inhibition increases lipid deposition in the liver and macrophage accumulation in HFD mice

After 2 weeks of HFD feeding, lipid deposition in the liver was not obviously altered compared with mice fed the control diet (Fig. S2a,b). The COMT inhibitor enhanced lipid deposition in the liver, and 2-ME ameliorated such COMT-inhibitor-increased fat deposition (Fig. S2c,d). HFD mice displayed insignificant F4/80 positive macrophage accumulation in the liver compared with control mice (Fig. S2e,f,m); COMT inhibitor treatment of the HFD mice resulted in remarkable accumulation (Fig. S2g,m). 2-ME treatment inhibited macrophage accumulation in COMT inhibitor-treated mice (Fig. S2h,m). Macrophage accumulation in the epididymal fat was increased in HFD mice (Fig. S2i,j,n). COMT-inhibitor treatment further enhanced macrophage accumulation in the epididymal fat and exhibited typical crown like structures (Fig. S2k,n); 2-ME inhibited macrophage accumulation (Fig. S2l,n). 2 weeks feeding of HFD increased the trend of the liver triglycerides level; COMT inhibitor treatment further exacerbates the level of liver triglycerides in the HFD fed mice; 2-ME injection displayed remarkable suppression in COMT inhibitor treated HFD fed mice (Fig. S3).

### Long-term treatment of 2-ME antagonizes glucose tolerance defects in insulin resistant HFD mice

**Figure 2:**
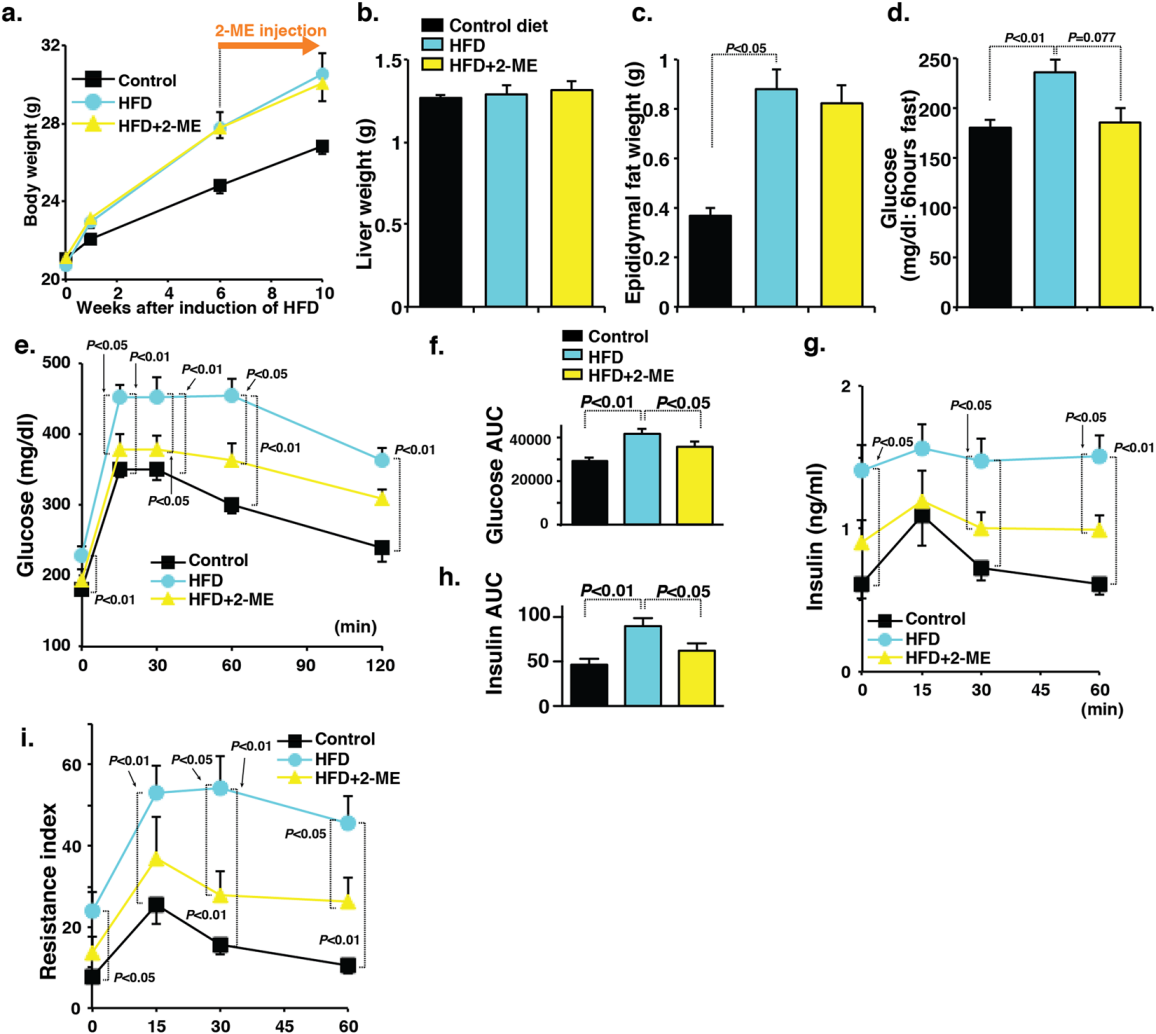
2-ME interventions for 4 weeks ameliorate glucose tolerance defects in chronic HFD induced-insulin-resistant mice. (**a**–**d**) Body weight, liver weight, epididymal weight and fasting blood glucose levels were analyzed in control, HFD, and 2-ME intervened HFD mice. N = 8 were analyzed in each group. (**e**) The IPGTT analysis. N = 8 from each group were analyzed. (**f**) Measurement of AUC value of glucose. (**g**) Insulin level. N = 8 were analyzed in each data set. (**h**) AUC value of insulin. (**i**) Insulin resistance index. The data in the figures are shown as the mean ± s.e.m. N = 8 were analyzed in each data set. The Mann-Whitney test was carried out to determine of statistical significance. Prism7.0 software was utilized for the statistical calculation. The mice fed the control diet are designated as “control”, whereas the mice fed the HFD are designated as “HFD”.

We next investigated whether 2-ME intervention could ameliorate glucose tolerance and associated defects in chronic HFD fed insulin-resistant mice. Six weeks after the initiation of the HFD, some mice were selected for a 2-ME intervention study for 4 weeks. Both the 2-ME-treated and untreated mice were sacrificed at 10 weeks after the initiation of HFD. HFD mice exhibited significant weight-gain compared with the control diet fed mice; 2-ME intervention did not cause a reduction in body weight (Fig. 2a). The liver weights remained unchanged in all of the groups (Fig. 2b). The weight of epididymal fat was heavier in the HFD mice; 2-ME intervention did not significantly reduce HFD-induced gain of epididymal fat weight (Fig. 2c). Six-hour fast blood glucose was higher in the HFD mice; 2-ME suppressed the trend of increasing blood glucose levels (*P* = 0.077) (Fig. 2d). The IPGTT data revealed that the HFD mice exhibited impaired glucose tolerance compared with the mice fed the control diet; 2-ME treatment resulted in a amelioration in glucose tolerance (Fig. 2e,f). The HFD mice also exhibited higher insulin levels compared with the control mice (Fig. 2g,h); 2-ME treatment suppressed the elevated level of insulin in HFD mice (Fig. 2g,h). The insulin resistance index estimation revealed that 2-ME remarkably ameliorated insulin resistance compared with the untreated HFD mice (Fig. 2i). Hepatic steatosis is evident in HFD mice compared with control mice (Fig. S4a,b); 2-ME rescued HFD-induced hepatic steatosis (Fig. S4c). As expected, the HFD mice exhibited macrophage accumulation in the epididymal fat tissue and crown-like structure formation (Fig. S4d,e,j); 2-ME intervention inhibited macrophage accumulation in the epididymal fat tissue (Fig. S4f,j). Similarly, 2-ME inhibited HFD-induced macrophage accumulation in the liver (Fig. S4g–i,k). 10 weeks feeding of HFD in mice caused significant increase in the level of liver triglycerides and 2-ME treatment suppressed the elevated level of triglycerides in the liver of HFD fed mice (Fig. S3).

### COMT inhibition was associated with glucose tolerance defects during gestation

**Figure 3:**
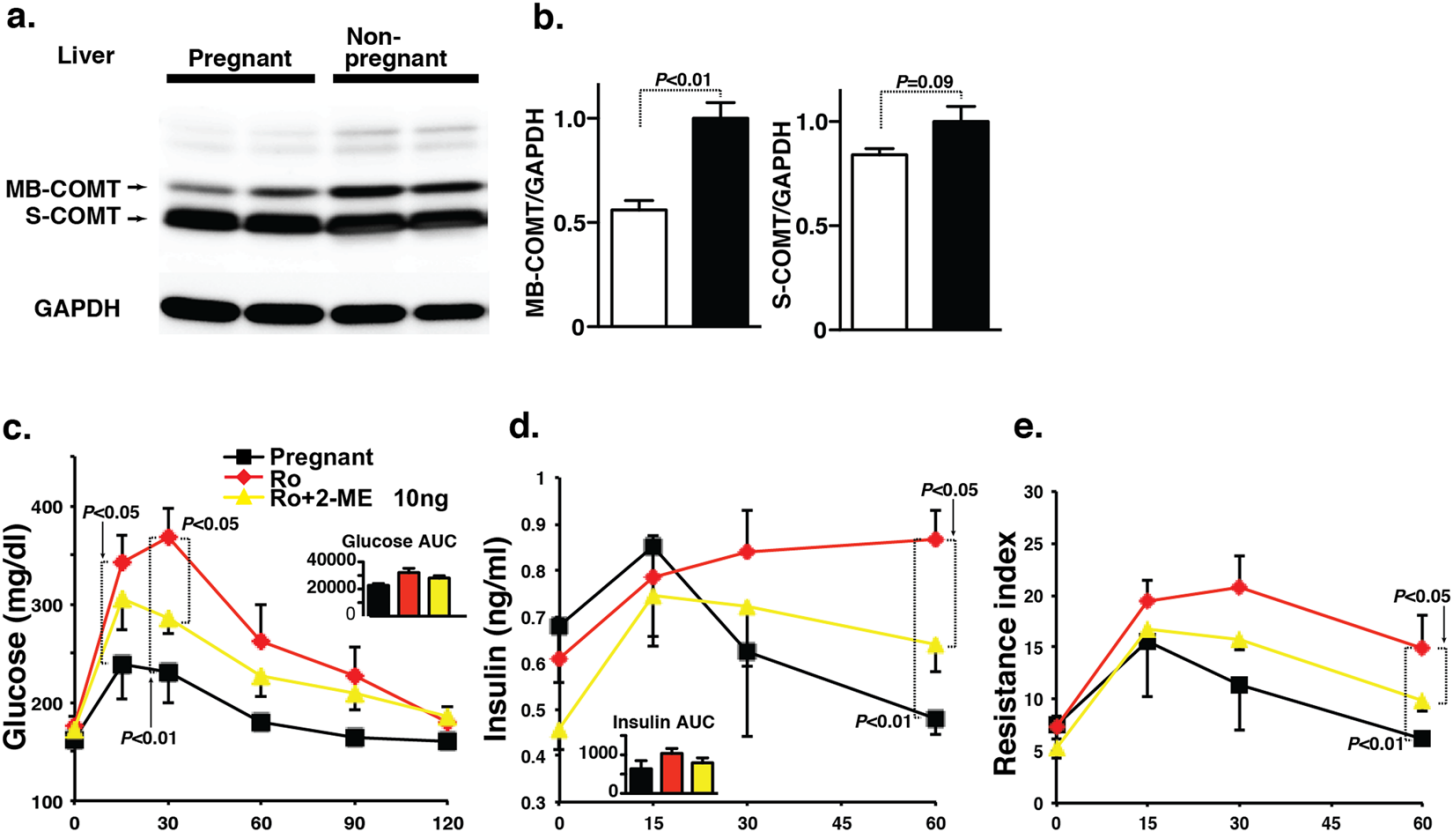
Inhibition of COMT during gestation contributes to glucose tolerance defects. (**a**) Western blot analysis of COMT protein level in the liver of pregnant and non-pregnant mice. Representative blot from 5 blots is shown. Cropped images were displayed and original blots are shown in the figure Supplementary 11. (**b**) Densitometric analysis of MB-COMT and S-COMT. Data were normalized to GAPDH. N = 5 were analyzed. (**c**) IPGTT analysis of pregnant, Ro-treatment and 2-ME (10 ng)-intervened Ro-treated mice groups at day 16 of pregnancy. Ro41-0960 treatment with or without 2-ME interventions were performed during day 10 to day 16 of the pregnant mice. Control N = 4, Ro41-0960 N = 3, and Ro41-0960 + 2-ME N = 3 were analyzed. (**d**) Insulin level was estimated at 0, 15-, 30-, 45- and 60-min time intervals. (**e**) Resistance index. N = 3 were analyzed in each mice group. The results are shown as the mean ± s.e.m. and are indicated in the figures. COMT inhibitor (Ro41-0960) was designated as Ro in the figure. The results in the figures are shown as the mean ± s.e.m. The Mann-Whitney test was carried out to determine of statistical significance. Prism7.0 software was utilized for the statistical calculation. Membranous COMT is designated as “MB-COMT”, whereas soluble COMT is designated as “S-COMT”.

We previously showed that pregnant COMT deficient mice exhibited a preeclampsia-like phenotype with significant diminished levels of 2-ME, and 2-ME injection cured all such phenotypes^3^. Preeclampsia is the condition associated with glucose tolerance defects^21^. Therefore, we analyzed the role of COMT deficiency in glucose tolerance of pregnant mice. In pregnant mice, liver COMT protein levels were significantly suppressed when compared to non-pregnant female mice fed normal chow, suggesting that pregnancy was the status associated with potential COMT deficiency in the liver (Fig. 3a,b). COMT deficiency has been associated with low 2-ME levels^3^. The glucose tolerance defects were evident in COMT-inhibitor-treated pregnant mice compared with the control pregnant mice (Fig. 3c). Glucose tolerance defects were associated with higher insulin levels and insulin resistance (Fig. 3d,e). In this experimental condition, 2-ME treatment (10 ng: same amount to cure preeclampsia phenotype in COMT deficient mice^3^) ameliorated such glucose tolerance defects, hyperinsulinemia, and insulin resistance (Fig. 3c–e). COMT inhibitor treatment displayed the deposition of triglycerides in the liver of pregnant mice whereas 2-ME intervention significantly suppressed the elevated level of liver triglycerides in COMT-inhibitor-treated pregnant mice (Fig. S3).

### Anti-diabetic mechanisms of metformin are associated with the restoration of COMT in liver

**Figure 4:**
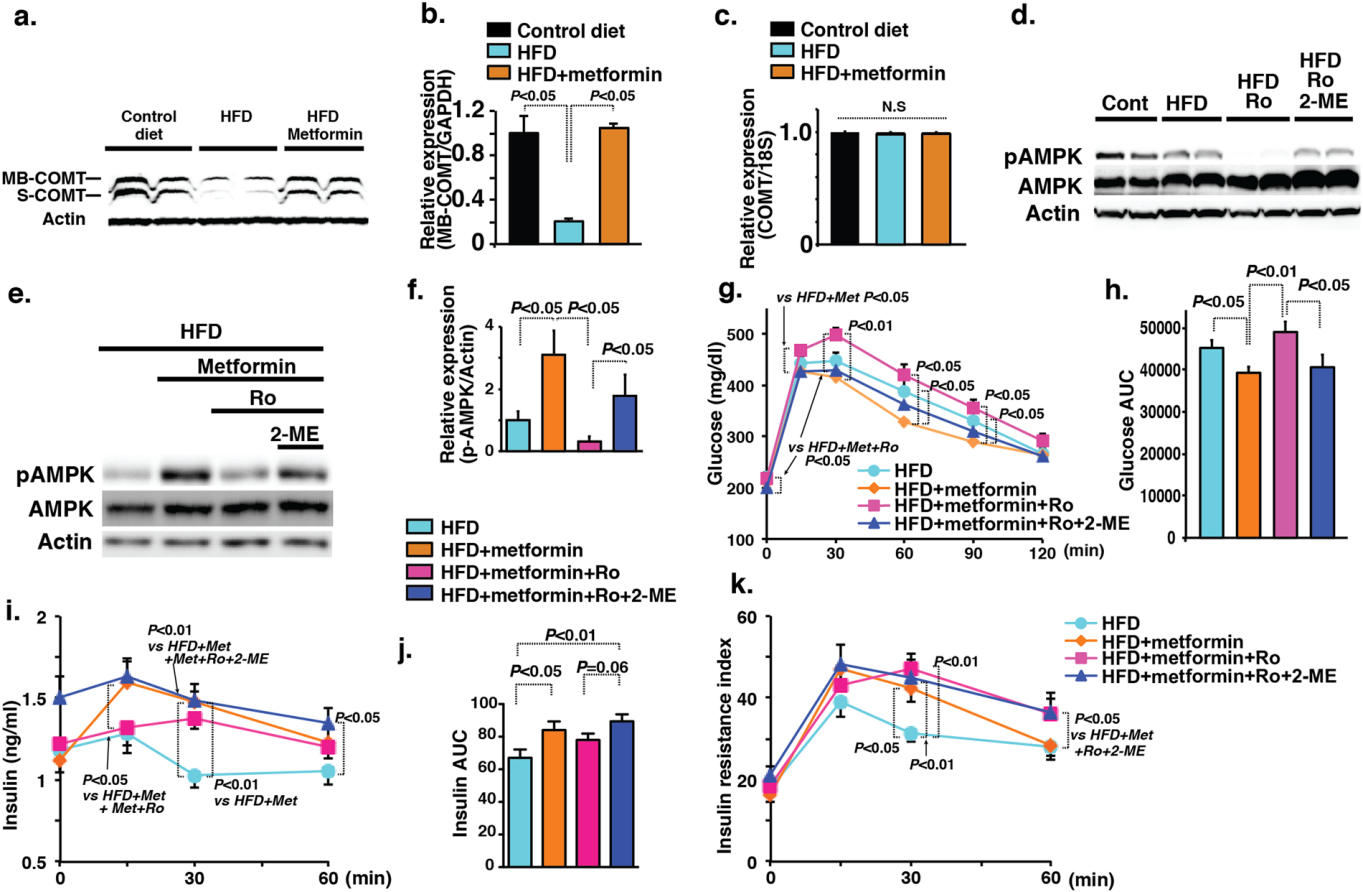
COMT is involved in the anti-diabetic action of metformin. (**a**,**b**) Western blot analysis of COMT protein level in the liver of control, HFD and metformin-treated HFD mice. Representative picture from 5 blots is shown. Cropped images were displayed and original blots are shown in the figure Supplementary 12. Densitometric data are normalized to actin. (**c**) COMT mRNA expression analysis. N = 3 were analyzed. (**d**) Representative image of western blot analysis of AMPK phosphorylation in the liver of control, HFD, HFD + Ro and Ro + metformin-treated HFD mice. N = 4 were analyzed per group. Cropped images were displayed and original blots are shown in the figure Supplementary 12. (**e**,**f**) Evaluation of AMPK phosphorylation by western blot analysis in the liver of metformin-treated HFD mice either administered Ro or Ro and 2-ME together. A representative image from 5 blots is shown. Cropped images were displayed and original blots are shown in the figure Supplementary 12. Densitometry data were normalized to total-AMPK. N = 4 were analyzed in each group. (**g**) IPGTT analysis. N = 9 were analyzed in each group. (**h**) AUC value of glucose. (**i**,**j**) Insulin level and AUC value of insulin. (**k**) Insulin resistance index. N = 8 or 9 were analyzed in each group. Data in the figures are expressed as the mean ± s.e.m. COMT inhibitor (Ro41-0960) was designated as Ro in the figure. Prism7.0 software was utilized for the statistical calculation. The Mann-Whitney test was carried out to determine of statistical significance.

We attempted to discover a potential drug to enhance the COMT- or 2-ME-mediated beneficial metabolic effects under HFD feeding. Because the similarity of the beneficial metabolic effects of 2-ME on chronic treatment on HFD mice, we focused on the biguanide class drug metformin. We found that metformin treatment caused a remarkable elevation in the liver COMT protein level of HFD mice (Fig. 4a,b) without an alteration in the mRNA level (Fig. 4c). Therefore, we hypothesized that COMT is essential for the anti-diabetic action of metformin. AMPK is the well-known target of metformin, and we found that COMT-inhibitor-treated HFD mice exhibited a significant suppression of AMPK phosphorylation in the liver, whereas 2-ME intervention caused restoration of the AMPK phosphorylation level (Fig. 4d). Similarly, the suppression of liver AMPK phosphorylation in COMT-inhibitor-treated pregnant mice was normalized by 2-ME treatment (Fig. S5a,b). When compared to HFD mice, metformin-treated mice exhibited enhanced AMPK phosphorylation (Fig. 4e,f). COMT inhibitor treatment abolished metformin-induced liver AMPK phosphorylation in mice, and 2-ME intervention partially restored AMPK phosphorylation in these mice (Fig. 4e,f). IPGTT analysis revealed that COMT inhibitor treatment abolished the blood glucose-lowering effects of metformin in HFD mice; 2-ME treatment restored blood glucose levels similar to levels after metformin treatment (Fig. 4g,h). Surprisingly, the plasma insulin levels were indeed increased by metformin compared with HFD alone (Fig. 4i,j). The COMT inhibitor suppressed insulin levels compared with metformin-treated HFD mice (Fig. 4i). 2-ME increased insulin levels in HFD metformin-COMT-inhibitor-treated mice (Fig. 4i,j). Indeed after a 2-week experimental period, metformin treatment did not inhibit insulin resistance (Fig. 4k), and neither the COMT inhibitor nor 2-ME treatment altered insulin resistance compared with metformin alone (Fig. 4k), suggesting that in this 2-week period the glucose-lowering effects of these compounds was mediated by altering insulin secretion but not by modifying the insulin resistance. Metformin treatment suppressed the trend of the liver triglycerides level; COMT inhibitor significantly elevated liver triglycerides levels in the metformin treated mice; 2-ME intervention significantly suppressed liver triglycerides levels (Fig. S3).

### Silencing of COMT *in vivo* mimics the metabolic defects associated with COMT insufficiency

**Figure 5:**
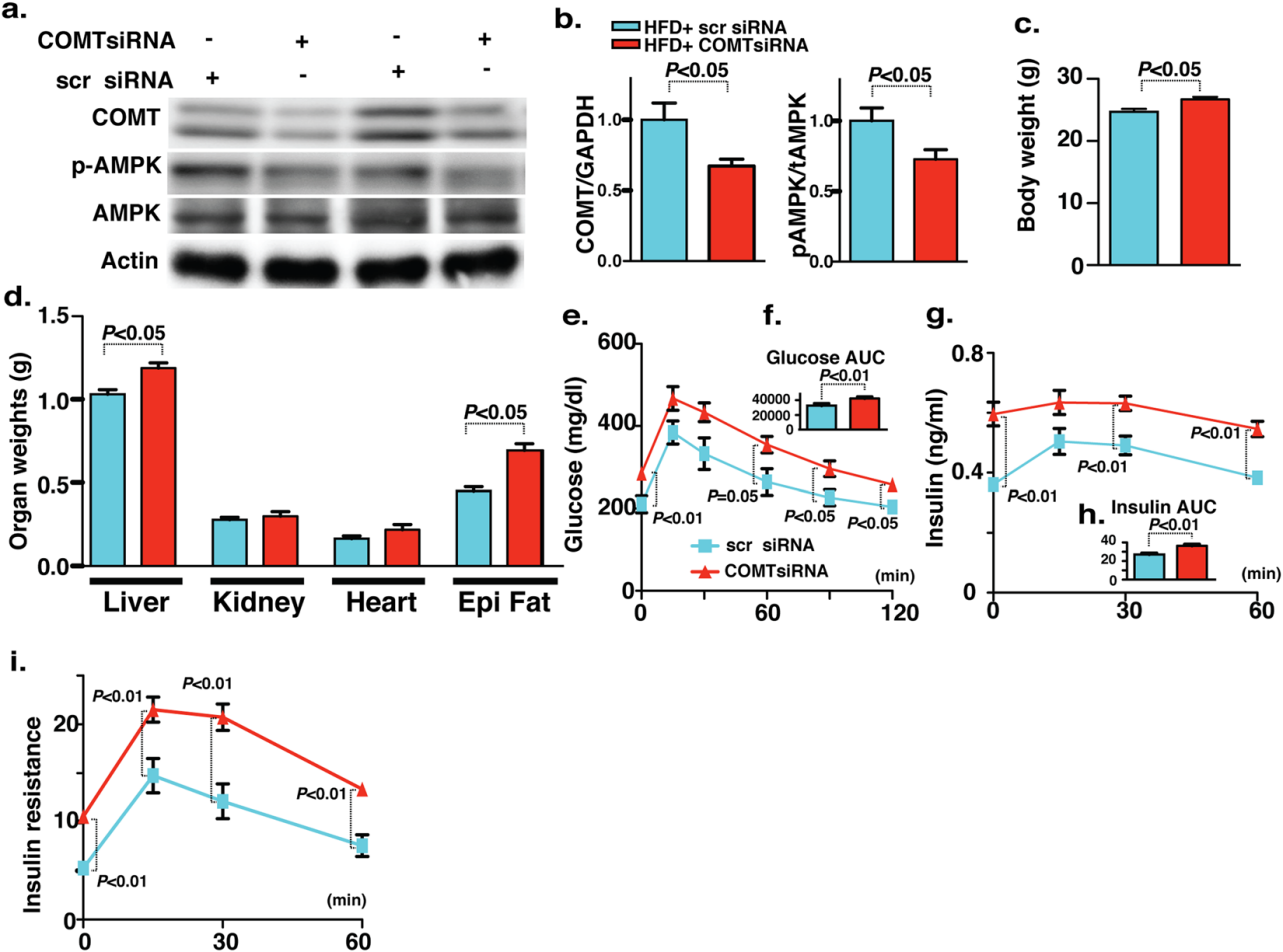
COMT siRNA-mediated silencing introduces the features of type 2 diabetes and metabolic syndrome in HFD mice. (**a**,**b**) Western blot analysis of COMT and AMPK phosphorylation in the liver of scramble and COMT siRNA-injected mice. Scramble and COMT siRNA were injected intraperitoneally once weekly for 3 weeks at a dose of 10 mg/kg body weight. A representative image from 6 blots is shown. Cropped images were displayed and original blots are shown in the figure Supplementary 13. Densitometric data analysis is normalized to Actin. N = 6 were analyzed in each data set. (**c**,**d**) Body weight and organ weight measurements in the scramble and COMT siRNA injected mice. N = 6 were analyzed. (**e**) IPGTT analysis. N = 6 were analyzed in each data set. (**f**) glucose AUC value. (**g**,**h**) Insulin value at different time intervals (0, 15, 30 and 60 min post glucose load) with AUC. N = 6 were analyzed in each data set. (**i**) Insulin resistance index. N = 6 were analyzed in each data set. Data in the graph are shown as the mean ± s.e.m. Scramble siRNA was designated as scr siRNA whereas COMT siRNA was designated as COMTsiRNA in the figure. Prism7.0 software was utilized for the statistical calculation. The Mann-Whitney test was carried out to determine of statistical significance.

To rule out the non-specific chemical compound effects of the COMT inhibitor, we tested whether gene silencing of COMT by siRNA could mimic the metabolic defects associated with the COMT inhibitor. We intraperitoneally injected either scramble or COMT specific siRNA once weekly for 3 weeks at a dose of 10 mg/kg BW. This procedure successfully inhibited liver COMT protein levels associated with the suppression of AMPK phosphorylation without alterations in the total AMPK level compared with the scramble siRNA-treated mice (Fig. 5a,b). Body (Fig. 5c), liver, and epididymal fat weights were heavier in the COMT siRNA-treated group compared with the scramble siRNA-treated mice (Fig. 5d). Injection of COMT siRNA into mice caused a significant elevation in the 6-hour fasting blood glucose compared with scramble siRNA injection (Fig. 5e). IPGTT analysis exhibited a remarkable exacerbation in the glucose intolerance in the COMT siRNA-treated HFD-fed mice compared with scramble siRNA-treated HFD-fed mice (Fig. 5e,f). An increase in the insulin level and insulin resistance was found in the COMT siRNA-treated mice compared with control mice (Fig. 5g–i). The COMT siRNA-treated HFD-fed mice showed excessive fat deposition in the liver and macrophage accumulation in the epididymal fat and liver as compared to the scramble siRNA-treated mice (Fig. S6a–h).

### Testosterone levels were not altered by either COMT deficiency or 2-ME intervention

2-ME is an endogenous metabolite of estradiol but has shown to be no affinity with estrogen receptors. Therefore theoretically 2-ME dose not display estrogenic effects. To make sure that 2-ME dose not affects androgen levels, we measured serum testosterone levels and as expected we did not find any difference in the level of testosterone by 2-ME treatment in each experiment (Fig. S7).

### 2-ME mimics PPARγ agonistic activity

2-ME has been known to exhibit structural similarity with PPARγ ligands^22^. Regard with this the normal pregnancy has shown to be high PPARγ activity in plasma^23^, however such pregnant-related increased activity of PPARγ was significantly diminished in preeclamptic women^24^. 2-ME has been shown to activate PPARγ^22, 25^ and we have also shown 2-ME suppressed angiotensin II type 1 receptor levels via PPARγ dependent manner^26^. To confirm whether such PPARγ agonistic activity of 2-ME was relevant in our experimental condition, we analyzed PPARγ levels and PPARγ target molecules in liver and epididymal fat of 2-week and 10-week protocol. In either liver or epididymal fat of high fat fed mice PPARγ protein levels were suppressed trend (in 2 weeks protocol COMT-inhibitor further suppressed) (Fig. S8) and 2-ME restored such levels (Fig. S8) as similar to PPARγ agonist reported elsewhere^27^. Also typical PPARγ target molecules such as CD36, FSP27, and PEX11a protein and mRNA levels exhibited similar trend in liver (Figs S8 and S9). Also in epididymal fat, we analyzed FABP4, FABP5, and LIPE mRNA expressions and found that except LIPE mRNA levels in 2-week protocol, all others suppressed in high fat (COMT-inhibitor further suppressed) and 2-ME restored (Fig. S9).

### 2-ME-induced augmentation of insulin secretion in MIN-6 cells

**Figure 6:**
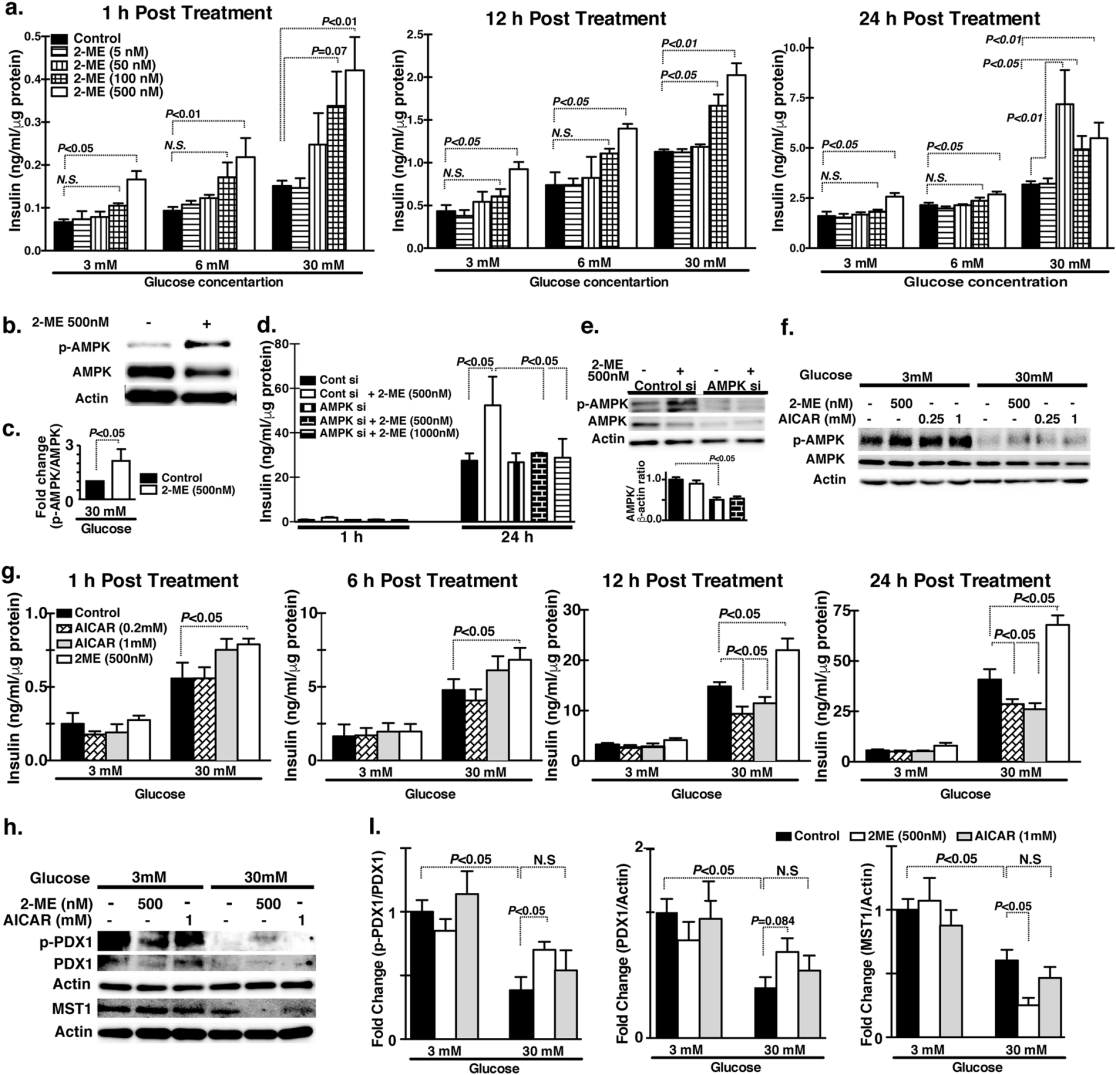
2-ME induced insulin secretion in MIN6 cells. (**a**) Time-dependent insulin secretion by 2-ME (5, 50, 100 and 500 nM concentrations) at 3 mM, 6 mM and 30 mM medium glucose concentrations. Insulin estimation by the ELISA method performed in triplicate. Three sets of independent experiments were performed. (**b**,**c**) Western blot data analysis of control and 2-ME-treated MIN6 cells. A representative image of 5 blots is shown. Cropped images were displayed and original blots are shown in the figure Supplementary 14. Densitometry data were normalized to β-actin. (**d**) Time-dependent insulin secretion by 2-ME in the scramble and AMPK siRNA transfected MIN6 cells. Scramble and AMPK siRNA were transfected using lipofectamine 2000 at 100 nM concentration in cells. Insulin estimation was performed in triplicate. Three sets of independent experiments were analyzed. (**e**) Western blot analysis of total AMPK and AMPK phosphorylation protein levels in the scramble and AMPK siRNA transfected MIN6 cells. A representative image from 5 blots is shown. Cropped images were displayed and original blots are shown in the figure Supplementary 14. Densitometry data normalized to β-actin. (**f**) Western blot analysis of total AMPK and AMPK phosphorylation after treatment with 2-ME and AICAR at 3 mM glucose and 30 mM glucose concentration. A representative image from 4 blots is shown. Cropped images were displayed and original blots are shown in the figure Supplementary 15. (**g**) Time-dependent insulin level in 2-ME and AICAR-treated cells in the 3 mM glucose and 30 mM glucose media concentration. Insulin estimation assays were performed in triplicate. (**h**,**i**) Western blot analysis of PDX1, PDX1 phosphorylation and MST1 in 2-ME and AICAR-treated cells under 3 mM and 30 mM of glucose concentration for 24 hours. A representative image of 4 blots is shown. Cropped images were displayed and original blots are shown in the figure Supplementary 16. Densitometry data were normalized to Actin. N = 4 were analyzed in each data set. The data in the graph are shown as the mean ± s.e.m. Prism3 software was utilized for the statistical calculation. The One way Anova (Tukey test) was carried out to determine of statistical significance.

AMPK activation in the liver by 2-ME may explain the anti-steatosis and anti-insulin resistance effects of 2-ME, but it was not clear how 2-ME enhanced the secretion of insulin in mice under certain conditions. To reveal the molecular mechanisms of the insulinotropic effects of 2-ME, we utilized the MIN-6 mouse β-cell line and investigated the 2-ME induced augmentation of insulin secretion and its association with AMPK phosphorylation. Indeed, 2-ME-treated mice exhibited enhanced phospho-AMPK immunoreactivity in the pancreatic islets compared with the COMT-inhibitor-treated mice, in which the immunoreactivity of phospho-AMPK was weak in the islets and also ductal cells, similar to the observation in the liver (Fig. S10a–h). 2-ME augmented insulin secretion both at low and high concentrations of glucose (Fig. 6a). In the 30-mM glucose condition, there was a trend of 2-ME dose dependency on the insulin secretion in MIN-6 cells (Fig. 6a). As expected, AMPK was activated by 2-ME treatment as a remarkable increase in the AMPK phosphorylation *in vitro* (Fig. 6b,c). Utilizing siRNA for AMPKα1 subunit, we confirmed that 2-ME did not increase the insulin levels in AMPK knockdown cells (Fig. 6d,e). Metformin alone increased neither the insulin secretion nor the phosphorylation of AMPK in MIN-6 β-cells (Fig. S9a–c). To confirm the role of AMPK phosphorylation on insulin secretion, we utilized AICAR, a known activator of AMPK phosphorylation, to treat MIN-6 cells. Similar to 2-ME, AICAR increased AMPK phosphorylation in MIN-6 cells (Fig. 6f); however, AICAR only showed a transient increased trend of insulin secretion in MIN-6 cells, and subsequently, AICAR-treated cells showed a suppression of insulin secretion 24 hours after incubation (Fig. 6g). To determine the difference between 2-ME and AICAR on insulin secretion, we analyzed molecules that are responsible for β-cell survival, such as PDX-1 and β-cell apoptosis such as MST-1^28^. Under the 3 mM glucose condition, these molecules were not altered in the presence of either 2-ME or AICAR compared with the control (Fig. 6h,i). Under the high glucose condition (30 mM), the phosphorylation of PDX-1, total PDX-1 and MST-1 were all suppressed compared with the 3 mM glucose condition, whereas 2-ME but not AICAR partially restored PDX-1 phosphorylation and PDX-1 protein levels. Additionally, 2-ME significantly suppressed MST-1 levels under the 30 mM glucose condition (Fig. 6h,i).

## Discussion

In this study, we focused on COMT deficiency-associated defects in glucose tolerance using short-/long-term HFD and pregnant mice. We found that HFD and pregnancy were associated with COMT suppression in the liver, suggesting that both of these conditions were prone for the onset of COMT deficiency. Further suppression of COMT in HFD-fed and in pregnant mice was associated with glucose tolerance defects; however, in contrast to our hypothesis, 2-ME displayed distinct roles in the amelioration of glucose tolerance, which included (1) amelioration of insulin resistance (long-term HFD and COMT inhibitor-treated control-fed male mice or pregnant mice) and (2) induction of insulin secretion. Upon observing a restoration of COMT protein levels in HFD mice after treatment with metformin, we found that COMT was a target of metformin. Surprisingly, metformin increased insulin levels in HFD mice, and such augmentation of insulin secretion by metformin was diminished in the presence of the COMT inhibitor. Our results indicated that metformin treatment induced the amelioration of glucose tolerance via the restoration of COMT protein levels and possibly an accumulation of methoxy-catechols, including 2-ME. We also confirmed our analysis directly by utilizing a specific COMT siRNA injection in HFD mice that displayed the characteristics of type 2 diabetes and metabolic syndrome. In addition to such diverse molecular mechanisms of glucose lowering, 2-ME displays anti-inflammatory effects that could affect improved insulin resistance^4^. Furthermore 2-ME displayed PPARγ agonist-like activities^22, 25, 26^ and restored the levels of HFD-suppressed PPARγ-target molecules in liver and epididymal fat. Collectively our data demonstrated that deficiency in COMT, either through chemical inhibitor or by gene silencing, exacerbated the glucose tolerance defects in HFD-fed mice. Finally, we found that 2-ME directly increased insulin secretion associated with AMPK phosphorylation, MST-1 suppression, and increased PDX-1 levels in MIN-6 cells. These findings shed new light on the biology of COMT deficiency and its pathological significance in the onset of metabolic diseases such as metabolic syndrome, type 2 diabetes, and preeclampsia.

We recognize several limitations of our study. First, we attempted to measure the concentration of 2-ME in our experimental animals using a commercially available kit; however, we could not monitor the level of 2-ME, possibly due to circulatory levels of 2-ME below the detectable range in our male mice. Therefore, we could not evaluate whether COMT deficiency in the liver is directly associated with the suppression of 2-ME in the plasma. Furthermore there was no way to evaluate the tissue 2-ME level precisely. Regard with this, we have already shown that in the pregnant status the condition associated with elevation of 2-ME, COMT deficiency was associated with notable suppression of 2-ME monitored by HPLC and LCMS/MS; even such experimental condition, we could not get difference in statistical significance in 2-ME level since 2-ME level were found significant lower in the mice when compared to human^3^. Second, we focused on 2-ME as a functional methoxy-catechol via COMT similar to the COMT-preeclampsia theory; however, it is possible that COMT deficiency under high fat diet feeding conditions could be associated with a wide variety of functional-methoxycatechols defects. Finally, we found that AMPK is likely the target of the COMT/2-ME system in association with the pharmacological function of metformin, although metformin exhibited AMPK-independent effects and AMPK-mediated insulin secretion is still controversial^29^. Our results suggest that the *in vivo* effects of metformin on enhanced insulin secretion was not a direct effect on the insulin-producing β cells. Further results suggest that 2-ME, but not AICAR, induced β cell survival signaling, such as MST-1 suppression and PDX-1 activation, under high glucose conditions in MIN-6 cells. Our results suggests that suppression of MST1 led to the induction of PDX1 phosphorylation and PDX1 protein level^28^ consistent with the report showing β cell trophic effects of 2-ME in diabetic db/db mice reported^30^. It is evident from our results, 2-ME did not induce insulin secretion in AMPK knockdown MIN-6 cells; however, AICAR-mediated significant activation of AMPK did not induce significant insulin secretion. Therefore, in our experiments, 2-ME required AMPK phosphorylation for insulin secretion; however, such AMPK phosphorylation by 2-ME is not sufficient for insulin secretion. Despite such limitations, our analysis clearly demonstrates that deficiencies in COMT and/or the methoxycatechols, such as 2-ME, are a potential therapeutic target to combat metabolic defects in certain environments such as HFD and pregnancy. The therapeutic effects of 2-ME on glucose tolerance defects, either via the amelioration of insulin resistance or the stimulation of insulin secretion, are likely dependent on the environments. Physiological rise in 2-ME concentration toward the term pregnancy is likely important for both the protection against preeclampsia^3^ and the adaptation for the higher insulin demand with resistance during pregnancy^31^.

Our findings would be relevant to various pathological conditions in human health. First of all, genetic variances of COMT associated with less enzymatic activity would be prone for the onset of diabetes, metabolic syndrome, and liver damage with certain metabolic insults associated with COMT suppression. Regard with this, pharmacological blockade of COMT by commercial available COMT-inhibitor entacapone has never been reported to associate with the onset of either diabetes or clinical apparent liver damage; this could be due to the short duration of action of entacapone (*t1/2* 0.4–0.7 hours)^32^. Indeed long acting COMT inhibitor tolcapone has been associated with severe liver injury^33, 34^. Also COMT is potentially suppressed by several endogenous and/or exogenous molecules, including polychlorinated biphenols (PCBs), dioxin, mercury, and S-adenosyl-L-homocysteine^35^. These molecules have shown to be associated with onset of metabolic defects and diabetes^36–39^. Further study would be required to analyze the interaction between genetic human COMT deficiency and environmental factors in the onset of metabolic defects with liver injuries.

In conclusion, deficiency in COMT is likely associated with metabolic defects in diverse physiological and pathological conditions, and such metabolic defects are partially explained by a deficiency in 2-ME. COMT deficiency could be the shared molecular mechanism between preeclampsia, the metabolic syndrome and type 2 diabetes.

## Materials and Methods

### Study Approval

The experiments in the methods sections are carried out in accordance with Kanazawa Medical University animal protocols (protocol number 2011–46, 2011–47, 2012–53, 2012–54, 2013–45, 2013–56, 2014–90, and 2015–100), approved by institutional animal care and use committee (IACUC). Authors confirm that all the experiments are performed in accordance to Japanese guidelines and regulations for scientific and ethical experimentation.

### Animal experiment protocol

Eight-week-old male C57/B6 mice were obtained from CLEA Japan, Inc. (Tokyo, Japan). The mice were fed with a control diet or HFD starting at 8 weeks of age. The metabolic status and histological/biochemical evaluations were performed at 2 or 10 weeks after the initiation of indicated diet. For the 2-week protocol, metformin (250 mg/kgBW/day: intraperitoneal), Ro41-0960 (25 mg/kgBW/day: subcutaneous) or 2-ME (10 ng/day: subcutaneous) were injected into the mice after the initiation of indicated diet. The interval of the drug or molecules was 1 week. For the 10-week protocol, 2-ME was injected at week 6 following the initiation of the indicated diet. 2-ME intervention was performed during the last 4 weeks.

### Pregnant phenotype analysis

Six-week-old female C57Bl6 mice were mated with male C57Bl6 mice. Successful mating was evaluated by the appearance of a vaginal plug, and the midday of the day when the vaginal plug was observed was considered to be 12 h after fertilization, embryonic day 0.5 (E0.5). The mice with a vaginal plug were placed in a different cage (maximum three pregnant mice to a cage) until being sacrificed at day 17 of gestation. Beginning at day 10 of the pregnancy, the mice were injected daily with Ro41-0960 (25 mg/kgBW) with or without 10 ng of 2-ME, or placebo (olive oil), subcutaneously. IPGTT was performed at day 16 of gestation. The mouse studies followed the Kanazawa Medical University Institutional Animal Care and Use Guidelines.

### Metabolic evaluation

An intraperitoneal glucose load (1 g/Kg body weight) was injected into each mouse. The blood glucose level of each animal was measured at 0, 15, 30, 60, 90 and 120 minutes post injection of glucose. The average decrease in the area under curve (AUC) in the experimental group compared with the control group was determined for statistical calculations. We measured insulin levels in the plasma of the mice via ELISA, (Morinaga Institute of Biological Science, Inc.). The insulin resistance index (IRI) was calculated by the method reported as IRI = Glucose(mmol/L) × Insulin(mU/L)/22.5)^20^.

### Oil red O staining and liver triglycerides measurement

Frozen sections were used for the evaluation of hepatic steatosis, and Oil Red-O staining was performed as previously described. Briefly, 8-µm sections were fixed in 10% formalin for 15 minutes, rinsed in distilled water, placed in 60% iso-propylene for 1 minutes, stained in Oil Red O solution (0.5 g Oil Red O dissolved in 100 ml iso-propylene) for 20 minutes in 37 °C, washed in 60% iso-propylene for 1 minute, then washed three times and mounted with 70% glycerin jelly. The red lipid droplets were observed under a microscope. Liver triglycerides were measures using triglycerides measuring kits (CELL BIOLABS INC.).

### Macrophage detection

Five-micron sections of frozen liver samples were used for macrophage labeling with F4/80. After blocking with PBS containing 2% BSA, the sections were incubated with anti-F4/80 (AbD Serotec, Oxford, UK) in PBS containing 2% BSA for 1 hour. The sections were washed three times and incubated with 1:200 diluted TRITC conjugated-secondary antibodies (Jackson Immunoresearch, West Grove, PA) at room temperature for 30 min. After washing 3 times and mounting with DAPI (Vector Laboratories, Inc. Burlingame, CA), F4/80 macrophages were analyzed under fluorescence microscopy and quantified by three independent fields in ×400 pictures in each mouse.

### *In-vivo* silencing studies by using COMT siRNA

C57BL6 male mice at 8 weeks of age (n = 8 in each group) were divided into two groups: HFD mice injected with scramble and HFD mice injected with COMT siRNA. A chemically modified HPLC purified COMT siRNA duplex (Sense strand 5′CACCAUGCAAACCACUACA(dTdT) and antisense strand 5′UGUAGUGGUUUGCAUGGUG(dTdT) as well as control siRNA duplex were synthesized and purchased from Bioneer Corporation (Daejeon, Korea). All of the oligos were dissolved in buffer (Atelo gene, Koken Co. Ltd. Japan) and injected intraperitoneally (100 μl) once weekly for 3 weeks at a dose of 10 mg/kg body weight. At the end of the experiment, an IPGTT was performed after a 6-h fast. All of the animals were given an intraperitoneal injection of glucose at a dose of 1 g/kg body weight, and the blood glucose levels were measured at 0, 15, 30, 60, 90 and 120 min. For insulin estimation, blood was withdrawn from the tail at 0, 30, 60 and 90 min.

### Serum testosterone level

Seurm testosterone level was evaluated with Testosterone ELISA kit (Enzo ADI-900–065, Enzo Life Sciences, Farmingdale, NY) following the manufacture’s protocol.

### Western blotting

Protein lysates were boiled in sodium dodecyl sulfate (SDS) sample buffer at 94 °C for 5 minutes. After centrifugation at 17,000 × *g* for 10 minutes at 4 °C, the supernatant was separated on 6% or 12% SDS-polyacrylamide gels, and blotted onto PVDF membranes (Immobilon, Bedford, MA) via the semidry method. After blocking with TBS-T (Tris buffered saline containing 0.05% Tween 20) containing 5% non-fat dry milk or BSA, the membranes were incubated with each primary antibody in TBST containing 5% bovine serum albumin at 4 °C overnight. The membranes were washed three times and incubated with 1:2000 diluted horseradish peroxide (HRP) conjugated-secondary antibodies (Cell Signaling Technology) at room temperature for 1 hour. The immunoreactive bands were detected with an enhanced chemiluminescence (ECL) detection system (Pierce Biotechnology, Rockford, IL).

### RNA isolation and qPCR

Total RNA was isolated from small pieces of liver or adipose tissue (10–20 mg) using Qiagen RNeasy Mini Kit (Qiagen, Hilden, Germany). Complementary DNA (cDNA) was generated by using the Super script (Invitrogen, Carlsbad, CA). qPCRs were performed in a 7900HT Fast real-time PCR system (Life technologies) using SYBR Green fluorescence(miScript SYBR Green PCR Kit, Qiagen) with 2ng of cDNA and quantified using the delta–delta-cycle threshold (Ct) method(ΔΔCt). All experiments were performed in triplicate and β-actin was utilized as an internal control. The mature sequences of specific primers (Table S1) were designed by Hokkaido System Science Co. (Hokkaido, Japan).

### Antibodies

Anti-COMT polyclonal antibody (C6870), anti-actin polycloncal antibody (A5060), and anti-GAPDH polyclonal antibody (G9545) were purchased from Sigma-Aldrich (St. Lous, MO). Anti-PDX1 (D59H3), anti-MST-1 (3682), phospho Thr172-AMPKα (40Η9), anti-AMPKα(D63G4) rabbit monoclonal antibodies were purchased from Cell Signaling (Danvers, MA). Anti-phospho Thr11-PDX1 polyclonal antibody (PA5-13046) was purchased from Thermo Fisher Scientific (Waltham, MA). Anti-PPARγ rabbit polyclonal antibody (ab209350), anti-CD36 rabbit monoclonal antibody (ab133625), anti-FSP27 mouse monoclonal antibody (ab77115), and anti-PEX11a rabbit polyclonal antibody (ab104959) were purchased from Abcam (Cambridge, UK).

### Cell culture and insulin secretion assay

MIN6 cells^40, 41^, kindly gifted from Dr. Dan Kawamoari at Osaka University, were routinely maintained in Dulbecco’s modified Eagle’s medium (DMEM) containing 25 mM glucose, supplemented with 10% fetal calf serum and 2 mM L-glutamine. The insulin secretion assays were performed by previously standardized procedures following manufacture’s instruction.

### Statistical analysis

The data were expressed as the means ± S.E.M. For the comparison among the multiple groups, one way ANOVA followed by Tukey’s test was used. For the comparison for 2 groups, Mann-Whitney test for analysis was used. *P* < 0.05 was recognized as statistical significance. GraphPad prism 7.0 was used for statistical analysis.

### Data availability statement

Authors declare that all data is available.

## Electronic supplementary material

supplementary info 1
