# Supplementary material for: Deficiency in catechol-o-methyltransferase is linked to a disruption of glucose homeostasis in mice
Source: Sci Rep. 2017 Aug 11;7:7927. doi: 10.1038/s41598-017-08513-w (PMC5554180; doi:10.1038/s41598-017-08513-w)
Supplement: Supplementary file 1 — supplementary info 1 [file 41598_2017_8513_MOESM1_ESM.pdf]

Deficiency in catechol-o-methyltransferase is linked to a disruption of glucose homeostasis in mice

Megumi Kanasaki[1†], Swayam Prakash Srivastava[1†], Fan Yang[1], Ling Xu[1], Sumiyo Kudoh[4], Munehiro Kitada[1,5], Norikazu Ueki[2], Hyoh Kim[3], Li jinpeng[1], Satoru Takeda[2], Keizo Kanasaki*[1,5], Daisuke Koya*[1,5]

Affiliations:

1. Department of Diabetology & Endocrinology, Kanazawa Medical University, Ishikawa, Japan
2. Department of Obstetrics and Gynecology, Juntendo University Faculty of Medicine, Tokyo, Japan..
3. Department of Medicine, Shiga University of Medical Science, Otsu, Shiga, Japan.
4. Department of General Medicine, Kanazawa Medical University, Ishikawa, Japan
5. Division of Anticipatory Molecular Food Science and Technology, Medical Research Institute, Kanazawa Medical University, Uchinada, Ishikawa, Japan 920-0293

†These authors equally contributed in this project

***Correspondence:**

Keizo Kanasaki, M.D. Ph.D. E-mail: kkanasak@kanazawa-med.ac.jp,

or

Daisuke Koya, MD, PhD, E-mail: koya0516@kanazawa-med.ac.jp

Department of Diabetology & Endocrinology, Kanazawa Medical University, Uchinada, Ishikawa 920-0293, Japan, TEL: 81-76-286-2211(Ex3305), FAX: 81-76-286-6927

Supplementary Information

Supplementary Table1

Primer sequence

primer	forward	reverse
FABP4	5'-ATGTGCGACCAGTTTGTG-3'	5'-TTTGCCATCCCACTTCTG-3'
FABP5	5'-CCTGTCCAAAGTGATGATGG-3'	5'-CAGCATCAGGAGTGGGATG-3'
LIPE	5'-TCCAAGCAGGGCAAAGAAG-3'	5'-GTGTCATCGTGCGTAAATCC-3'
CD36	5'-TCCTCTGACATTTGCAGGTCTATC-3'	5'-AAAGGCATTGGCTGGAAGAA-3'
FSP27	5'-GACCTCCTGAACAAGGTCCA-3'	5'-TAGCTGGGCTCTCTTCTTGC-3'
PEX11a	5'-CCGACTTTTCAGAGCCACTC-3'	5'-CGGTTAGGTTGGCTAATGT-3'
β-actin	5'-TGGCACCCAGCACAATGAA-3'	5'-CTAAGTCATAGTCCGCCTAGAAGCA-3'

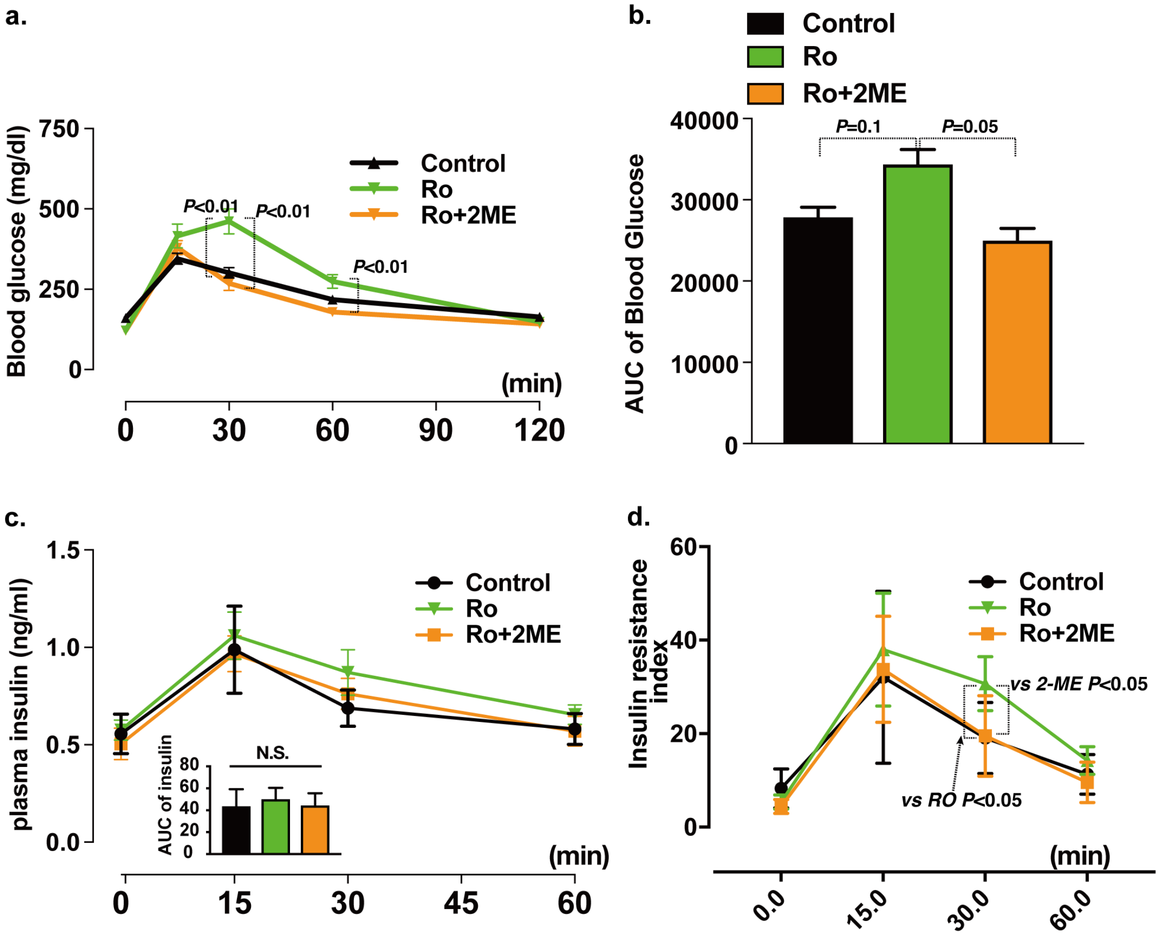

Figure S1: Effect of 2-ME on glucose homeostasis in control mice. a. Male C57Bl6 mice exposed to COMT inhibitor (Ro41-0960) with or without 2-ME treatment. IPGTT was performed as described in method section. **b:** AUC analysis of IPGTT in **a. c.** the levels of plasma insulin levels during IPGTT analysis. Inset of panel C indicated AUC analysis. **d.** Insulin resistance index calculated as method section. N=6 were analyzed for each group. One-way ANOVA followed by Tukey's test was performed for statistical significance. Data expressed as mean ±s.e.m. in the graph. COMT inhibitor was designated as Ro in the figure.

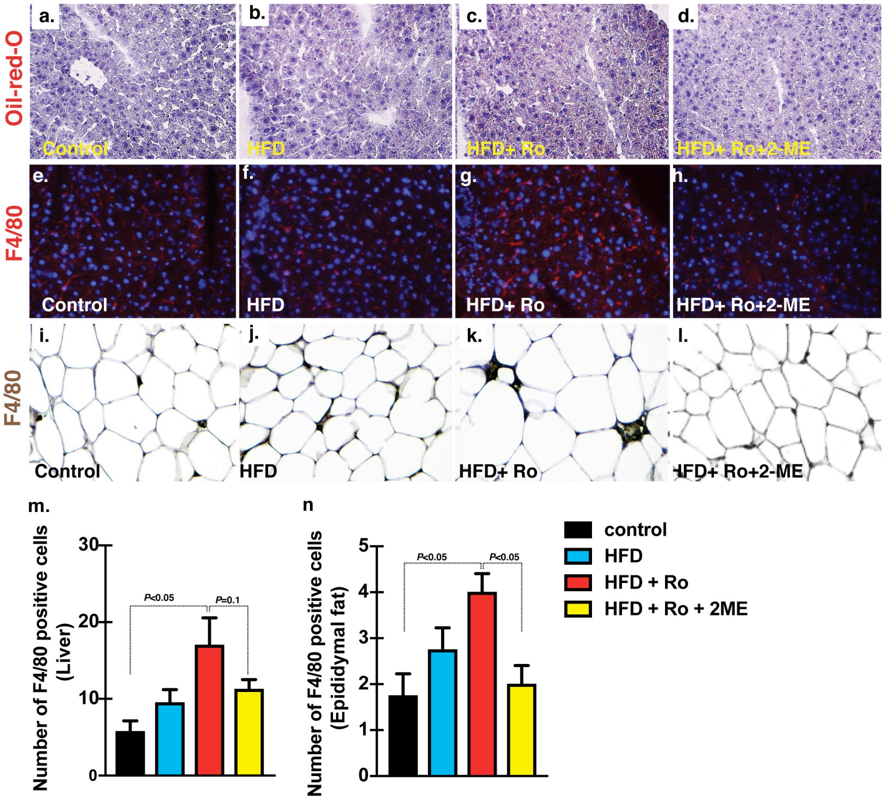

Figure S2. COMT inhibition is associated with increased lipid deposition in the livers of HFD mice. (a,b) Oil Red staining of the liver revealed that HFD mice had increased fat deposition in the liver. N=6 from each group were analyzed. **(c)** Treatment with Ro caused an increase in fat deposition in the liver. **(d)** 2-ME intervention in Ro-treated mice suppressed this fat accumulation, suggesting a protective role of COMT against hepatic steatosis. **(e,f)** Immunofluorescence analysis of macrophage marker (F4/80) in the liver revealed an increased expression in HFD mice compared with control mice. N=6 from each group were analyzed. **(g,h)** Ro treatment in HFD mice further increased macrophage infiltration in liver; 2-ME treatment in Ro-treated HFD mice inhibited this accumulation. Rhodamine (Red) shows F4/80 staining while DAPI (blue) shows nuclear staining. N=6 from each group were analyzed. **(i-l)** The F4/80 level was also increased in the epididymal tissue of Ro-treated mice, and 2ME intervention inhibited this macrophage accumulation. (**m,n**) quantification of macrophage in liver and epididymal fat. One-way ANOVA followed by Tukey's test was performed for statistical significance. The mice fed the control diet are designated as "control"; the mice fed the HFD are designated as "HFD", Ro treatment of HFD mice are designated as "HFD+Ro" and 2-ME treatment of HFD+ Ro mice are designated as "HFD+ Ro+2-ME". N=6 from each group were analyzed.

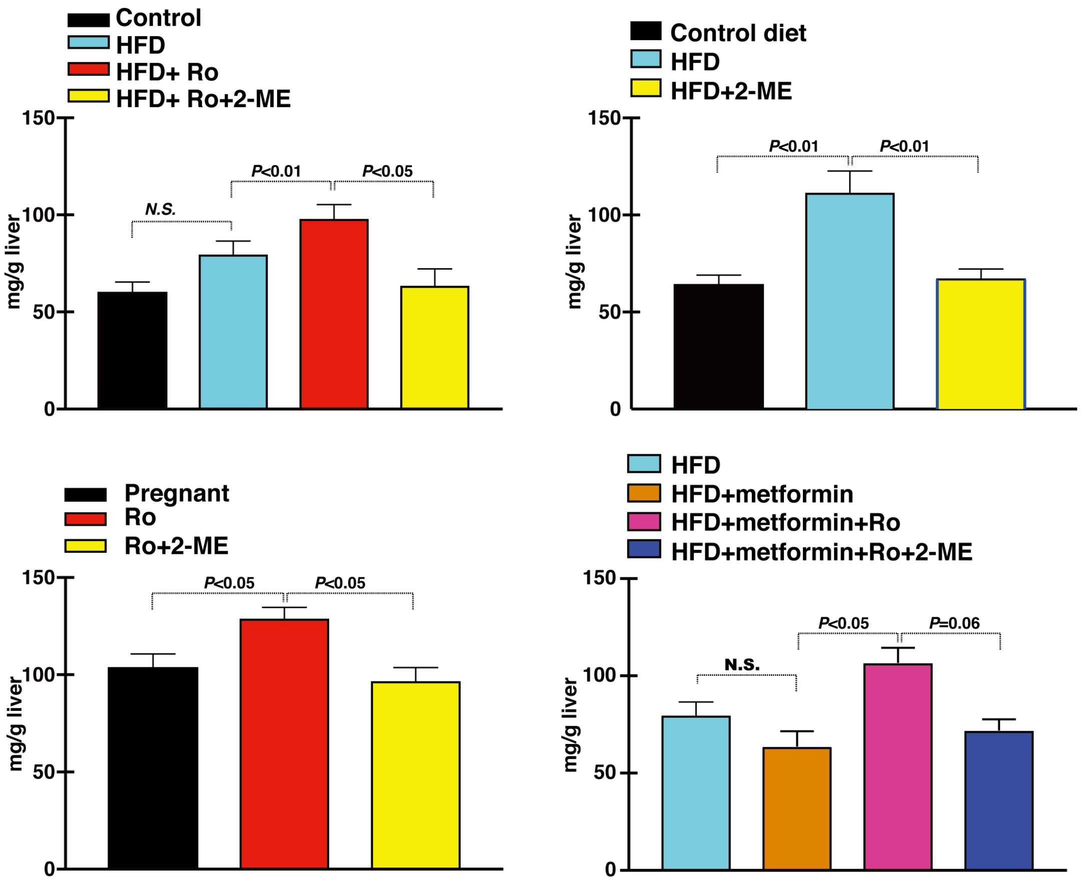

Figure S3. Effect of 2-ME on the liver triglycerides level. Liver homogenates were obtained from indicated groups of mice and triglycerides were measured (n=5~6) . One-way ANOVA Tukey's test was performed for statistical significance.

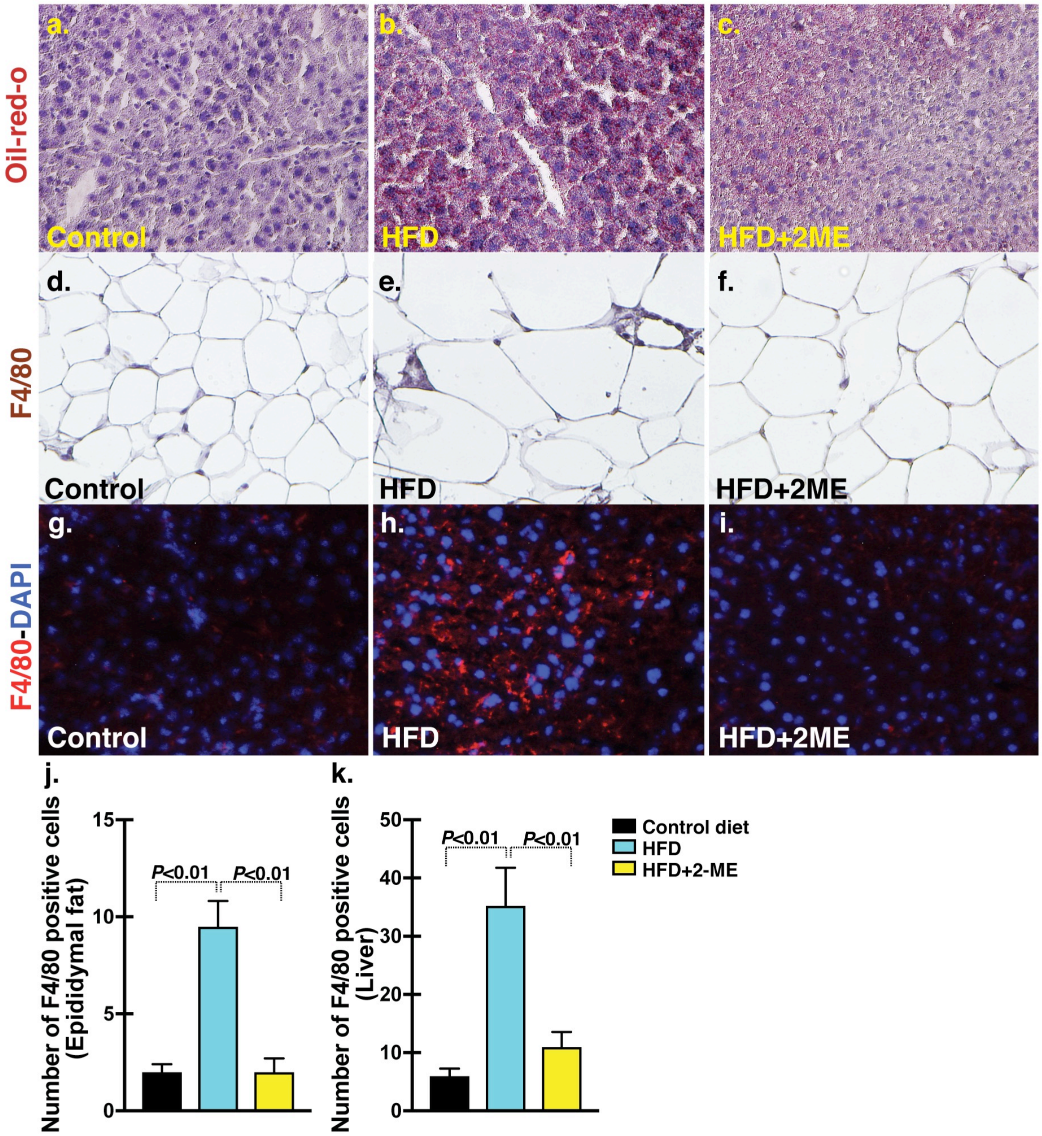

Figure S4. Chronic 2-ME treatment results in decreased lipid deposition and macrophage infiltration in HFD mice. (a-c) Oil Red staining in the liver of control, HFD, and 2-ME-treated HFD mice. N=6 from each group were analyzed. **(d-f)** F4/80 immuno-staining in the epididymal fat tissue of control ,HFD mice, and 2-ME treated HFD mice. N=6 from each group were analyzed. **(g-i)** Immunofluorescence analysis of F4/80 in the liver of control, HFD and 2-ME treated HFD mice. Rhodamine (Red) shows F4/80 staining while DAPI (blue) shows nuclear staining in the liver. (**j,k**) quantification of macrophage in epididymal fat and liver. N=6 from each group were analyzed. One-way ANOVA followed by Tukey's test was performed for statistical significance.

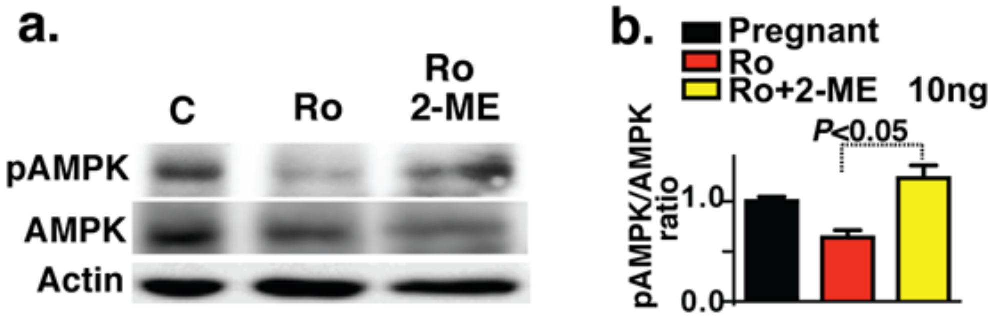

Figure S5. Suppression of liver AMPK activation in pregnant mice treated with COMT inhibitor.

(a) Western blot analysis of AMPK phosphorylation and total AMPK in the liver. Pregnant mice were injected with COMT inhibitor with or without 2-ME 10ng. Representative picture from 4 blots is shown. Cropped images were displayed and original blots are shown in the figure supplementary 17. **(b)** Densitometric data were normalized to β-actin. Data in the figures are expressed as the mean±s.e.m. N=6 were analyzed in each group. COMT inhibitor (Ro41-0960) was designated as Ro in the figure. The Mann-Whitney test was carried out to determine of statistical significance.

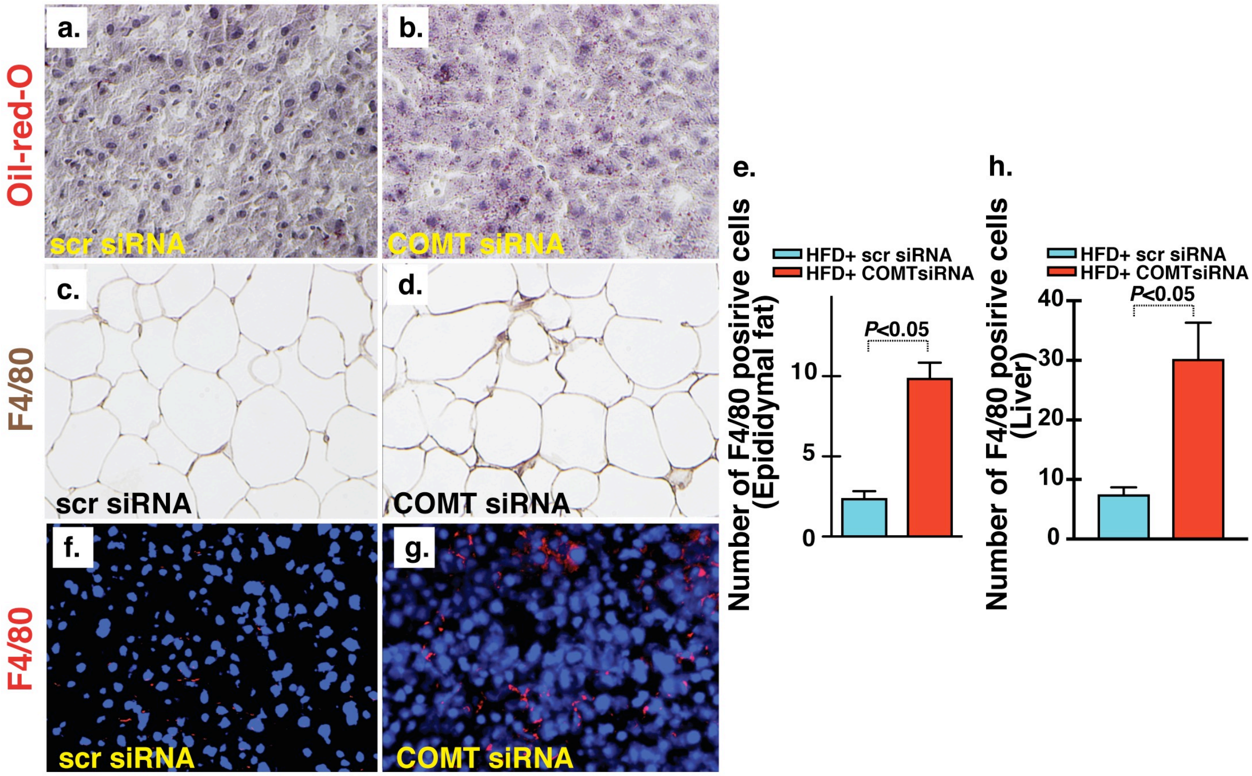

Figure S6. COMT siRNA knockdown in mice results in increased lipid deposition in the liver and increased macrophage infiltration in epididymal fat. (a,b) Oil red staining in the liver of COMT siRNA-treated mice and scramble siRNA-treated mice. N=5 were analyzed. **(c-e)** Immunohistochemical analysis of F4/80 in the epididymal fat of COMT siRNA-treated mice and scramble siRNA-treated mice. N=5 were analyzed. **(f,g)** Immunofluorescence analysis of F4/80 in liver of COMT siRNA and scramble siRNA treated mice. N=5 were analyzed. The data in the graph are shown as mean±s.e.m. Scramble siRNA was designated as scr siRNA whereas COMT siRNA was designated as COMT siRNA in the figure. (**e,h**) quantification of macrophage in epididymal fat and liver. The Mann-Whitney test was carried out to determine of statistical significance.

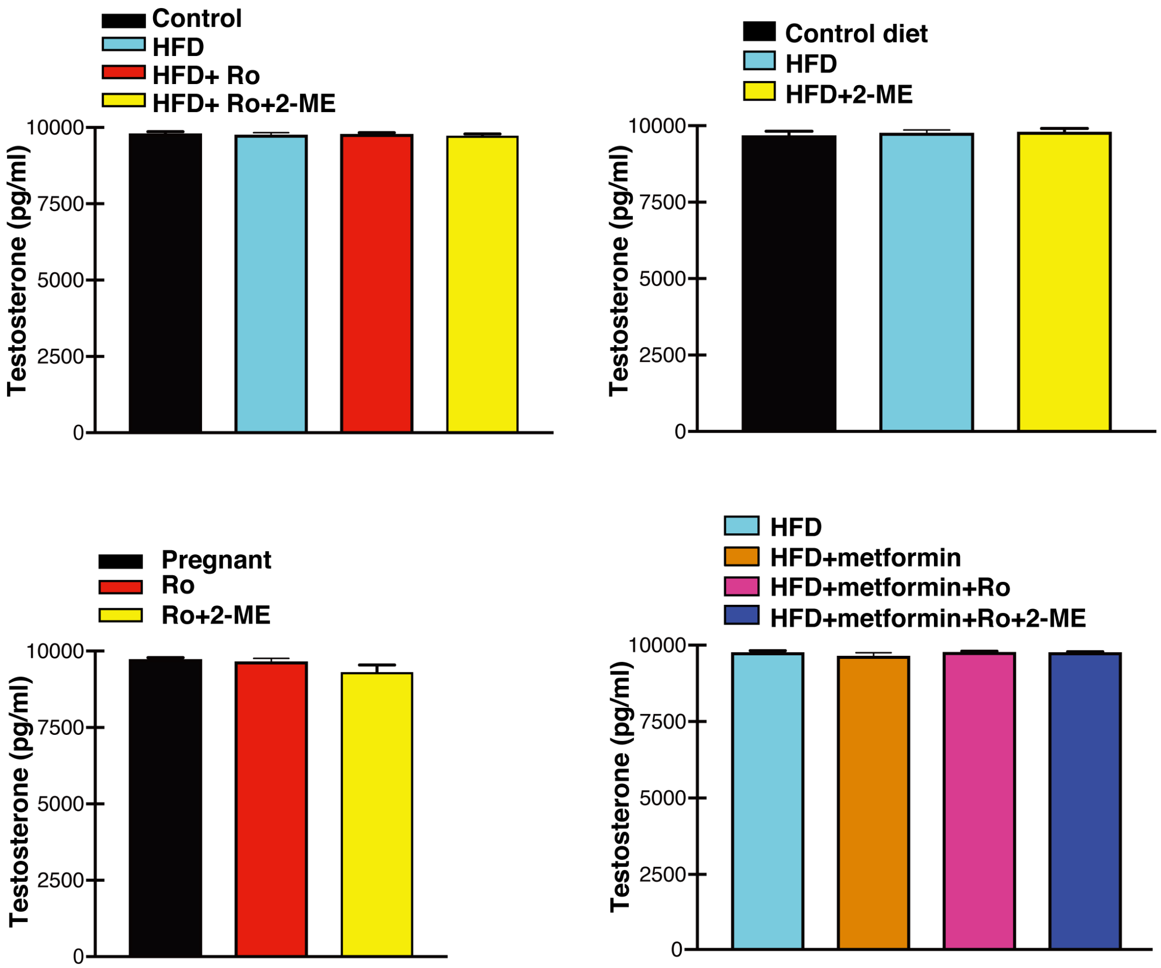

Figure S7. **Effect of 2-ME on testosterone level.** Plasma from each group of mice (n=5) was evaluated for levels of testosterone. Data in the figures are expressed as the mean±s.e.m.

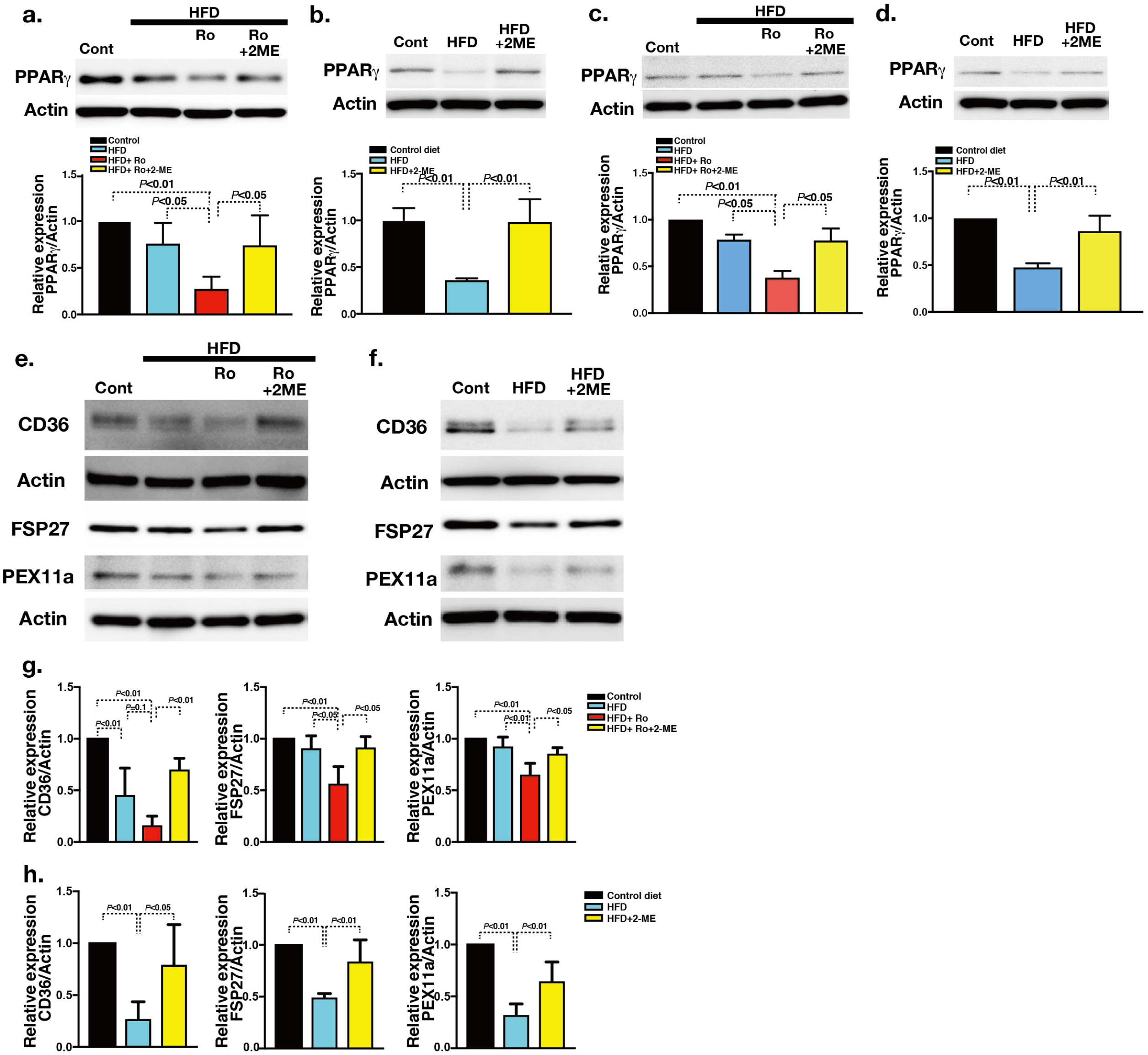

Figure S8. 2-ME exhibits PPARγ activity. (a,b) Liver and (c,d) epididymal fat PPARγ protein levels were analyzed in 2 weeks protocol (a, c) and 10 weeks protocol (b, d). Densitometric analysis was performed as relative expression to actin. n=3 for 2 weeks protocol (a,c) and n=4 for 10 weeks protocol (b,d). (e-f) Western blot analysis for PPARγ target molecules in liver. The representative blot images of 2 weeks (e) and 10 weeks (f) protocol were shown. (g, h) Densitometric analysis was performed as relative expression to actin. n=3 for 2 weeks protocol (g) and n=4 for 10 weeks protocol (h). COMT inhibitor (Ro41-0960) was designated as Ro in the figure. Data in the figures are expressed as the mean±s.e.m. One-way ANOVA followed by Tukey's test was performed for statistical significance.

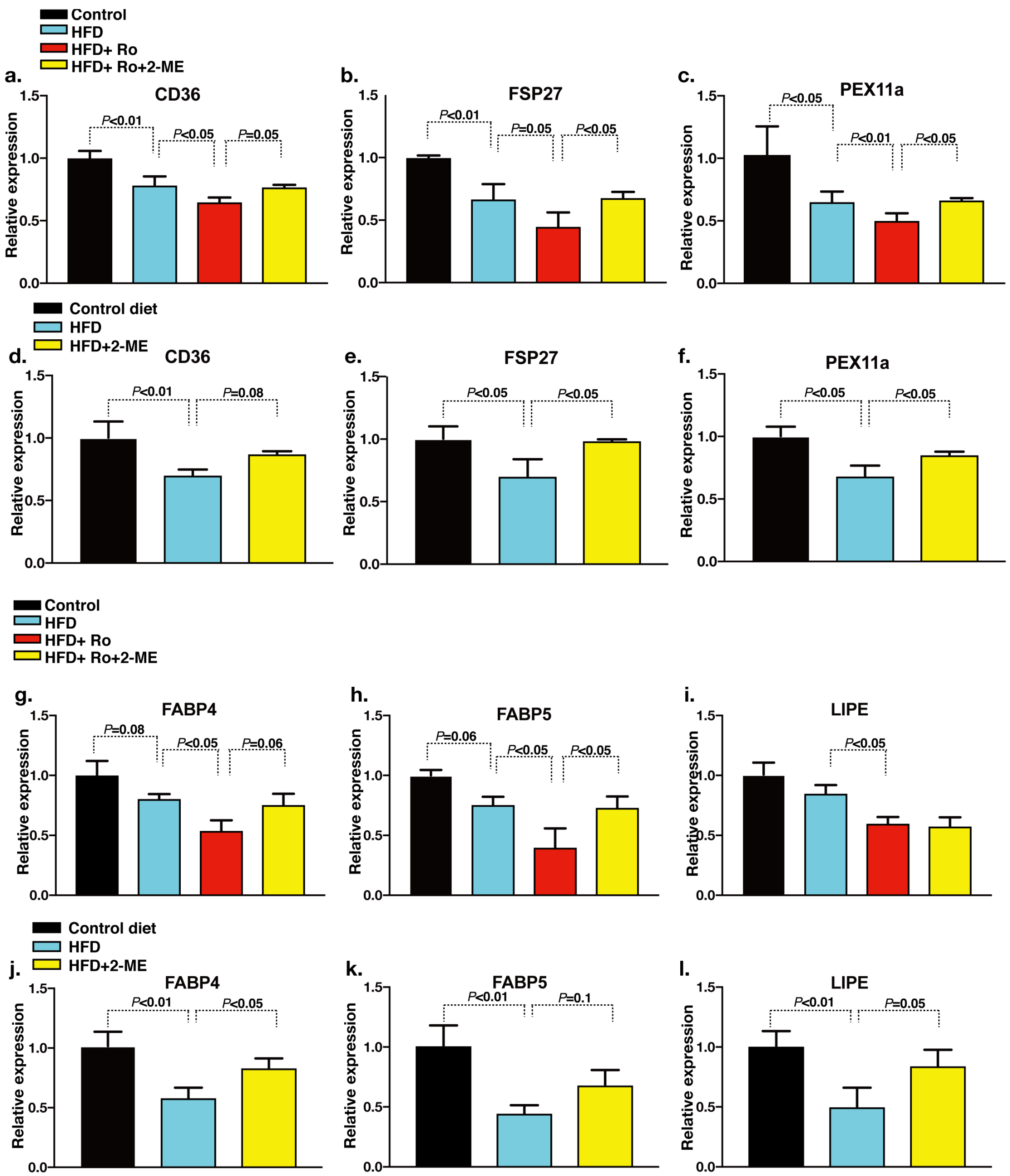

Figure S9 qPCR analysis for PPARγ target genes expression in liver and epididymal fat. qPCR analysis for indicated genes in liver (a-f) and epididymal fat (g-l) were shown. Data in the figures are expressed as the mean±s.e.m. One-way ANOVA followed by Tukey's test was performed for statistical significance.

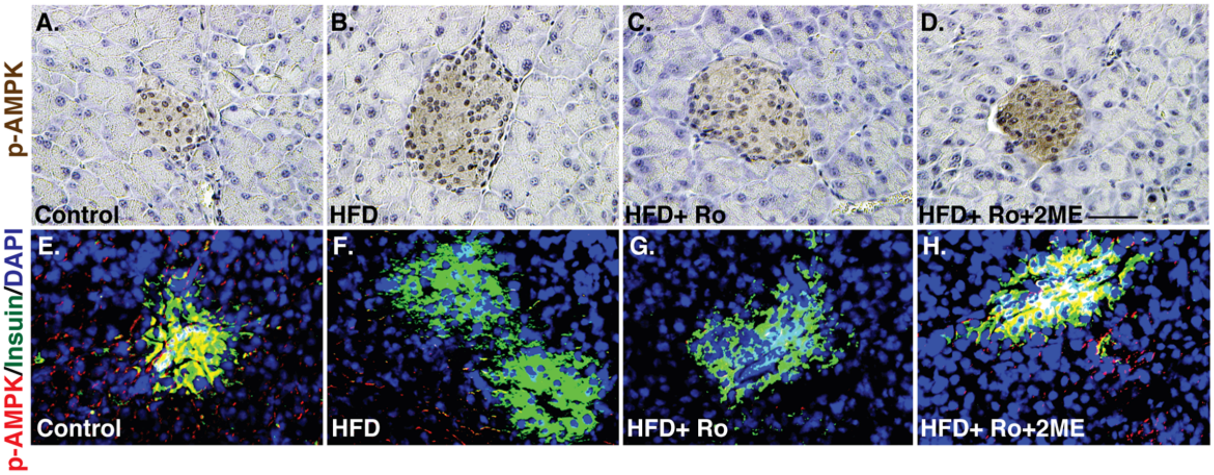

Figure S10. COMT inhibition is associated with decreased AMPK phosphorylation in the islets of HFD mice that is restored upon 2-ME intervention. (a-d) Immunohistochemical analysis of p-AMPK in the pancreatic islets reveals a remarkable increase in the 2-ME-intervened Ro-treated HFD mice compared with Ro-treated HFD mice. n=6 from each group were analyzed. **(e-h)** Dual-staining immunofluorescence analysis in the pancreatic islets of control, HFD, HFD+Ro- and HFD+ Ro+2-ME-treated mice. FITC (green) has shown insulin staining; rhodamine (red) shows p-AMPK staining while DAPI (blue) shows nuclear staining. The dual immuno-labeling (insulin/p-AMPK/DAPI) displays decreased staining in HFD mice, where Ro treatment in HFD mice did not result in remarkable alterations when compared with HFD mice. However, 2-ME intervention in Ro-treated HFD mice displayed remarkable dual staining. n=6 from each group were analyzed.

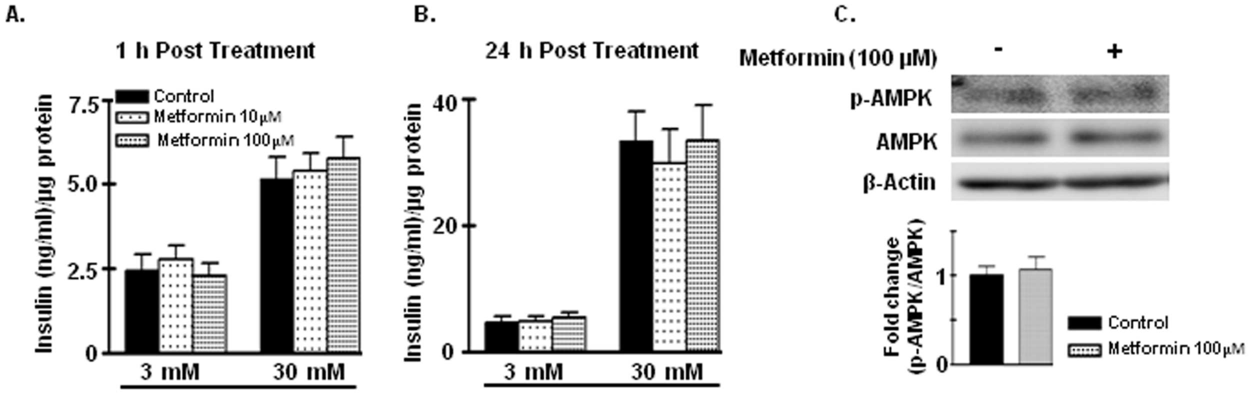

Fig. S11 Metformin treatment caused neither any remarkable alteration on the insulin secretion nor on the phosphorylation level of AMPK in MIN6 cells. (a) Insulin estimation at 1 hour post treatment of metformin on 10 and 100 µM metformin. Three independent set were performed. **(b)** Insulin estimation at 24 hour post treatment of metformin on 10 and 100 µM metformin. Three independent set were performed. **(c)** Western blot analysis of p-AMPK and AMPK. Protein samples were harvested from the 30 mM glucose treated MIN6 cells in absence and presence of metformin (100µM) by using RIPA buffer. Representative picture from 3 blots is shown. Cropped images were displayed and original blots are shown in the figure supplementary 18.

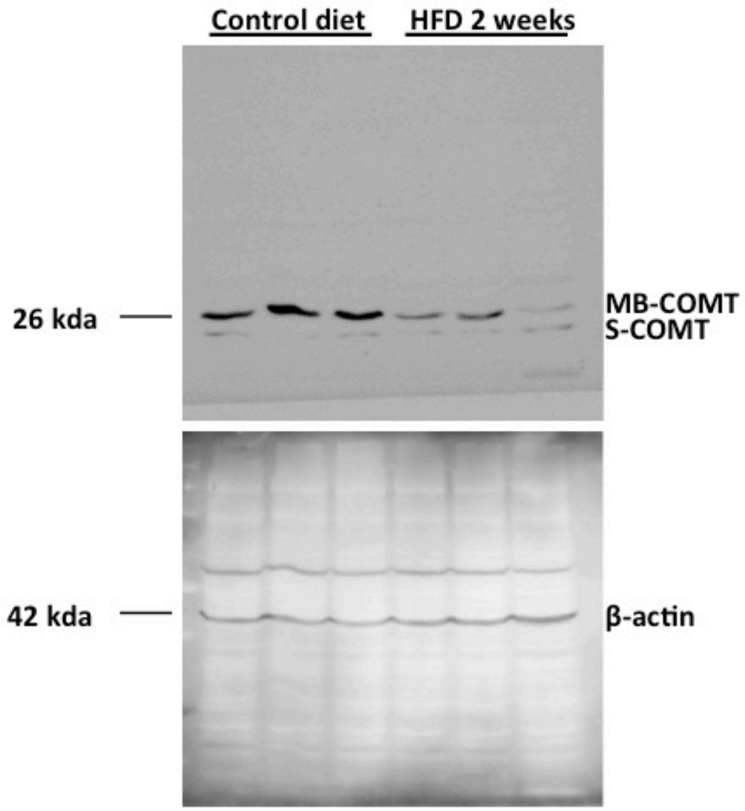

Fig. S12. Original blot for Fig 1a

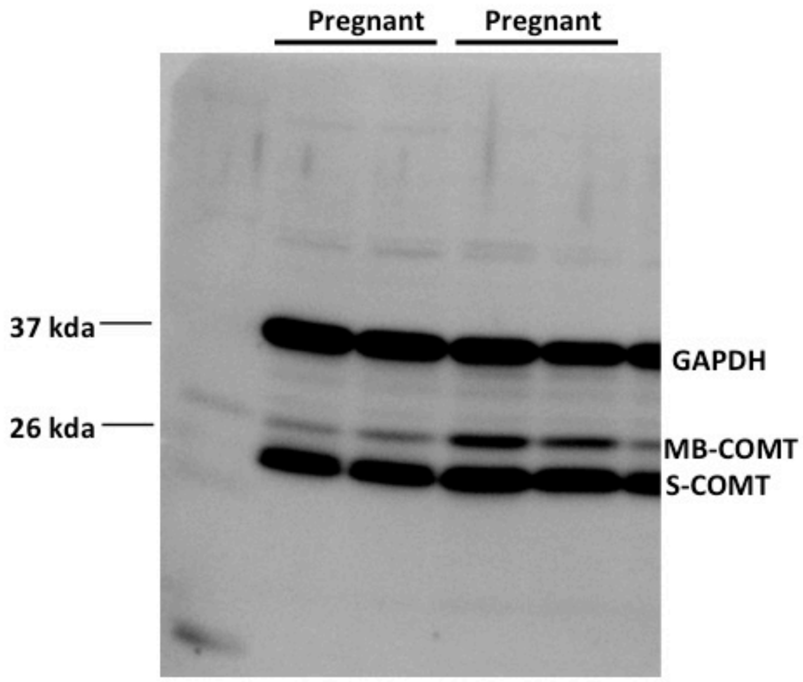

Fig. S13. Original blot for Fig 3a

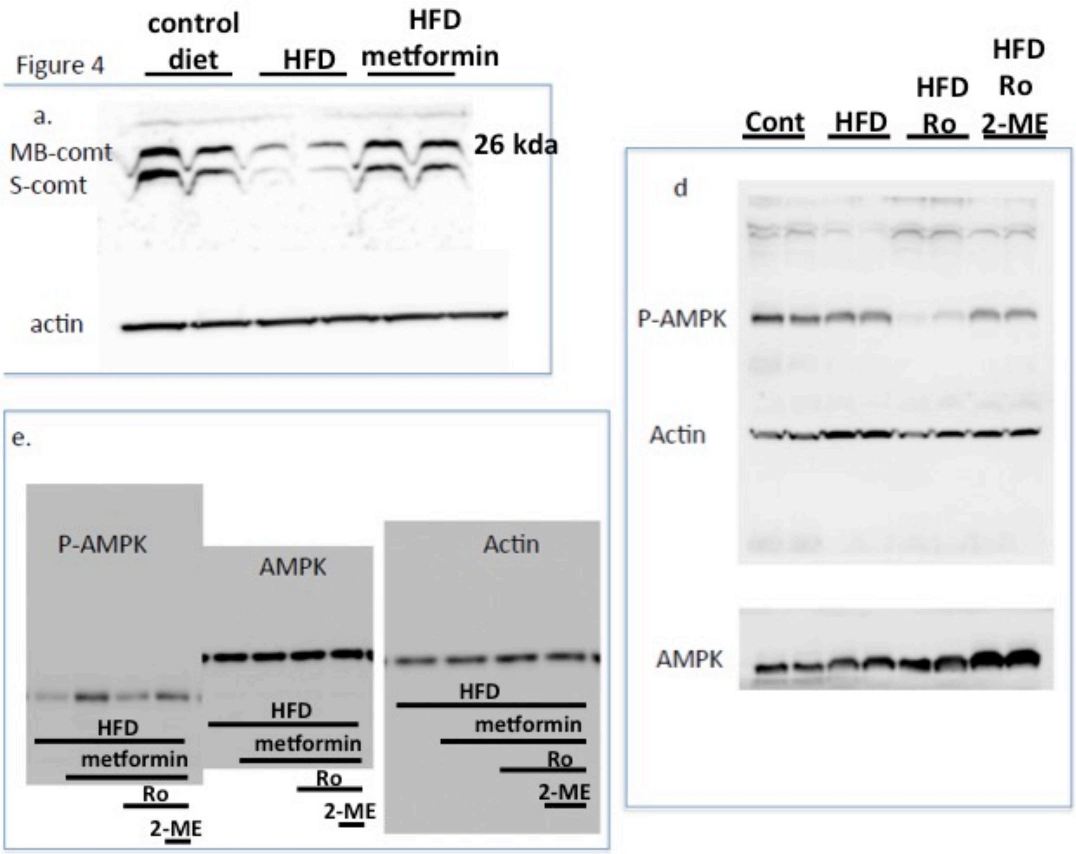

Fig. S14. Original blot for Fg 4 a, d, and e

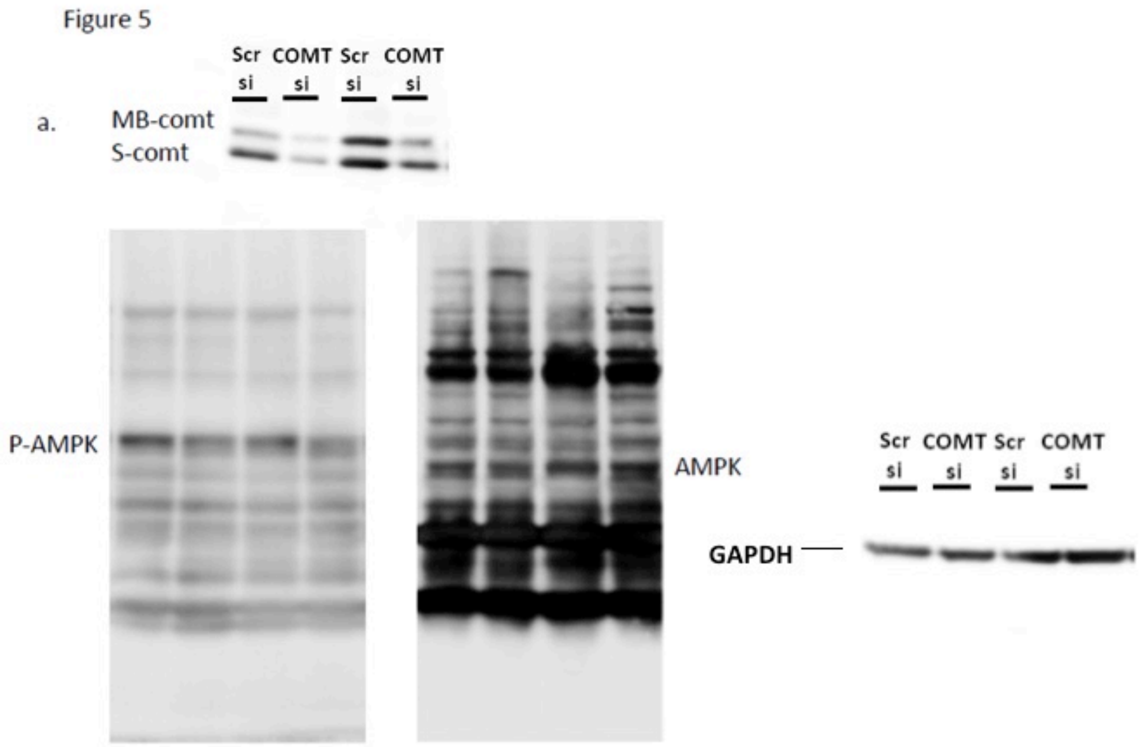

Fig. S15. Original blot for Fig 5a

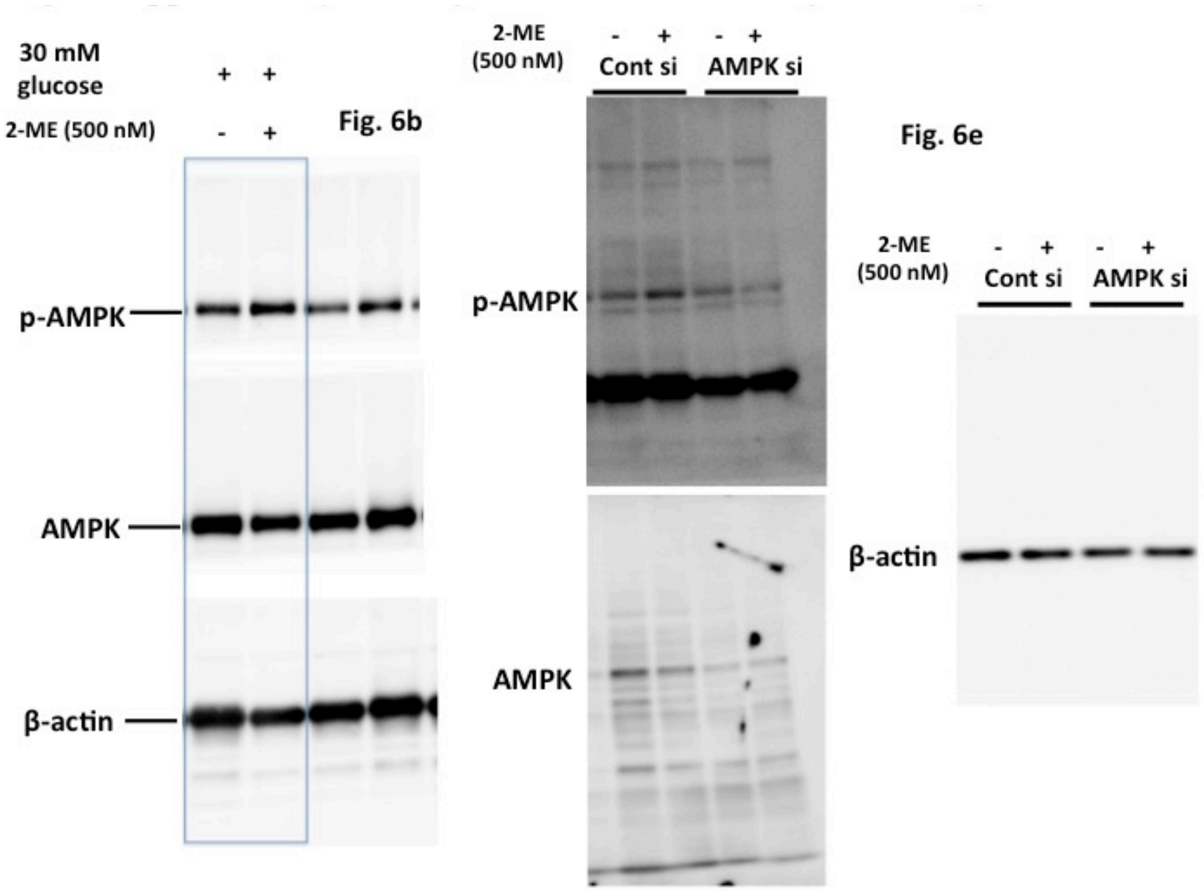

Fig. S16. Original blot for Fig 6 b and e

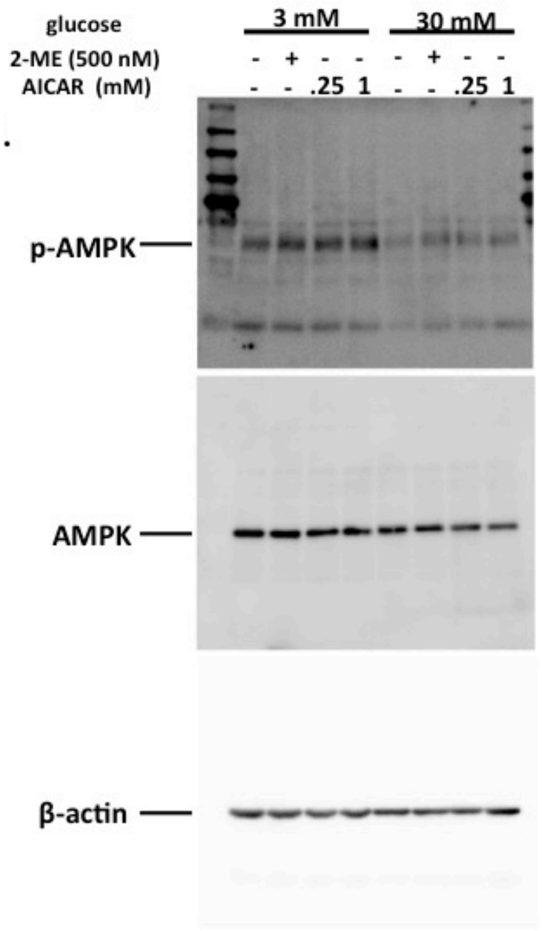

Fig. S17. Original blot for Fig 6f

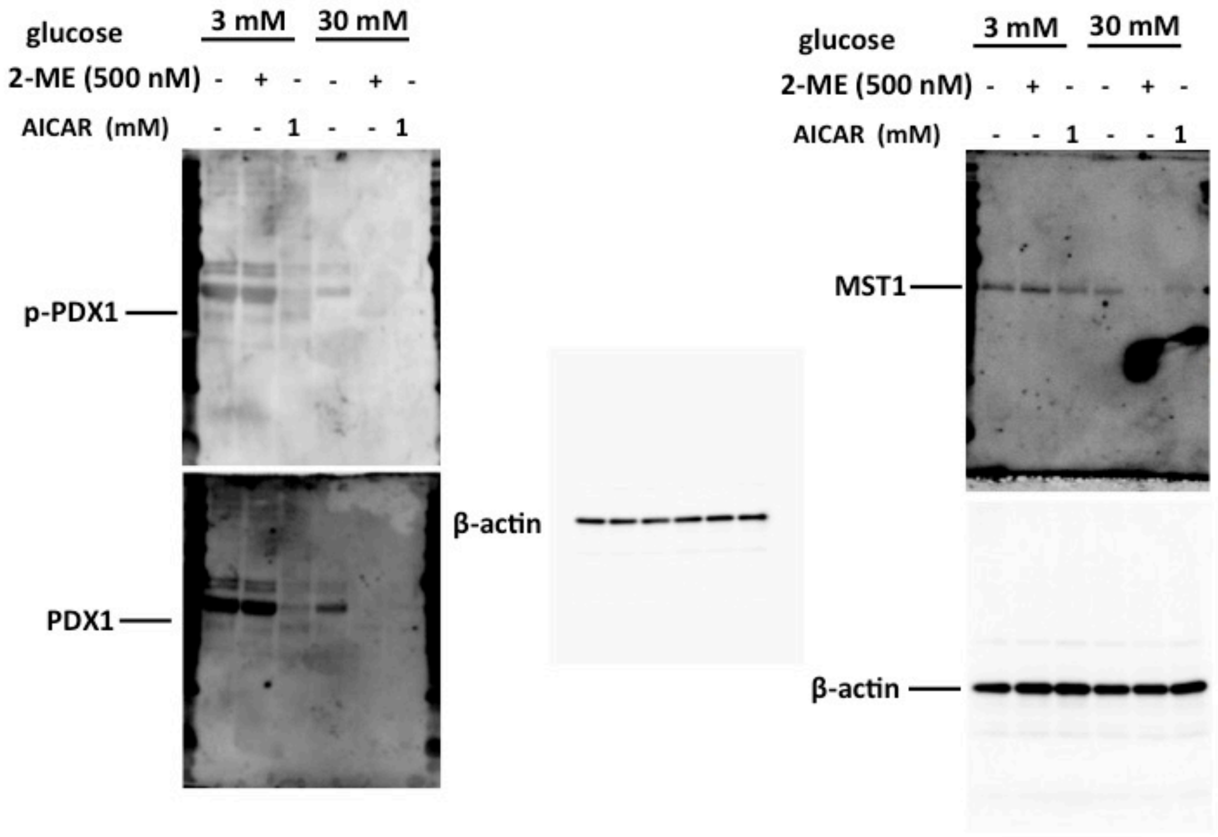

Fig. S18. Original blot for Fig 6h

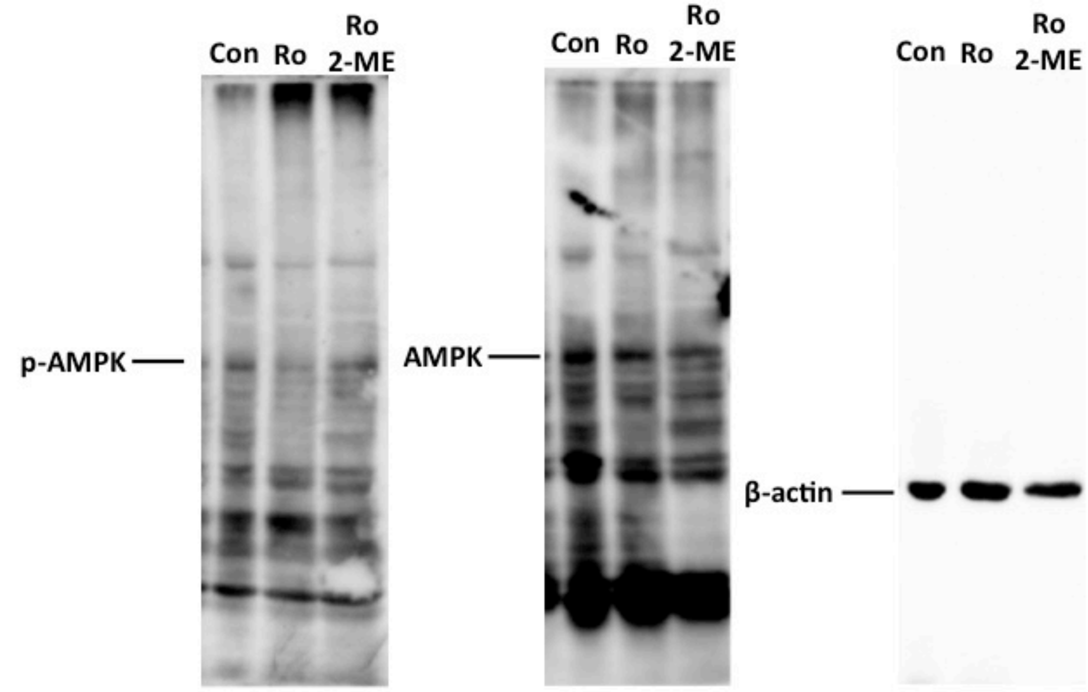

Fig. S19. Original blot for Fig S5

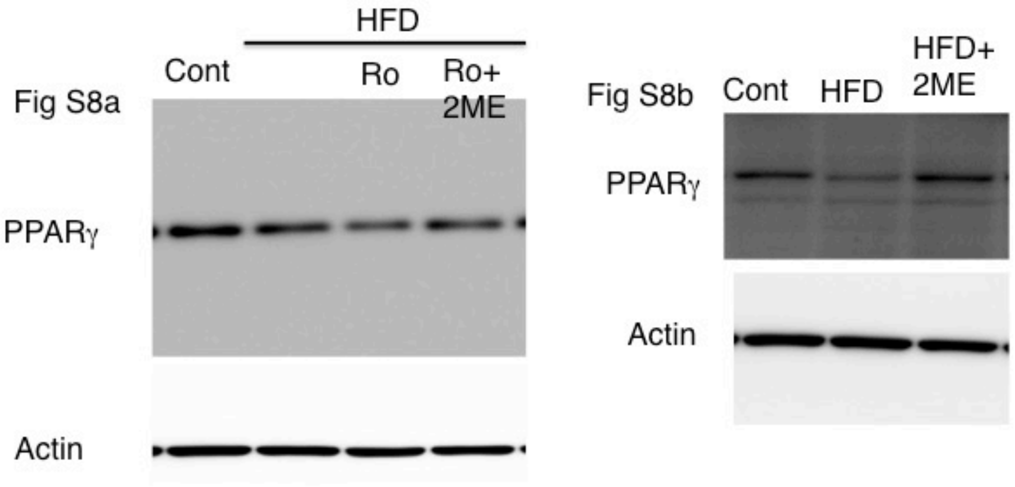

Fig. S20. Original blot for Fig S8 a and b

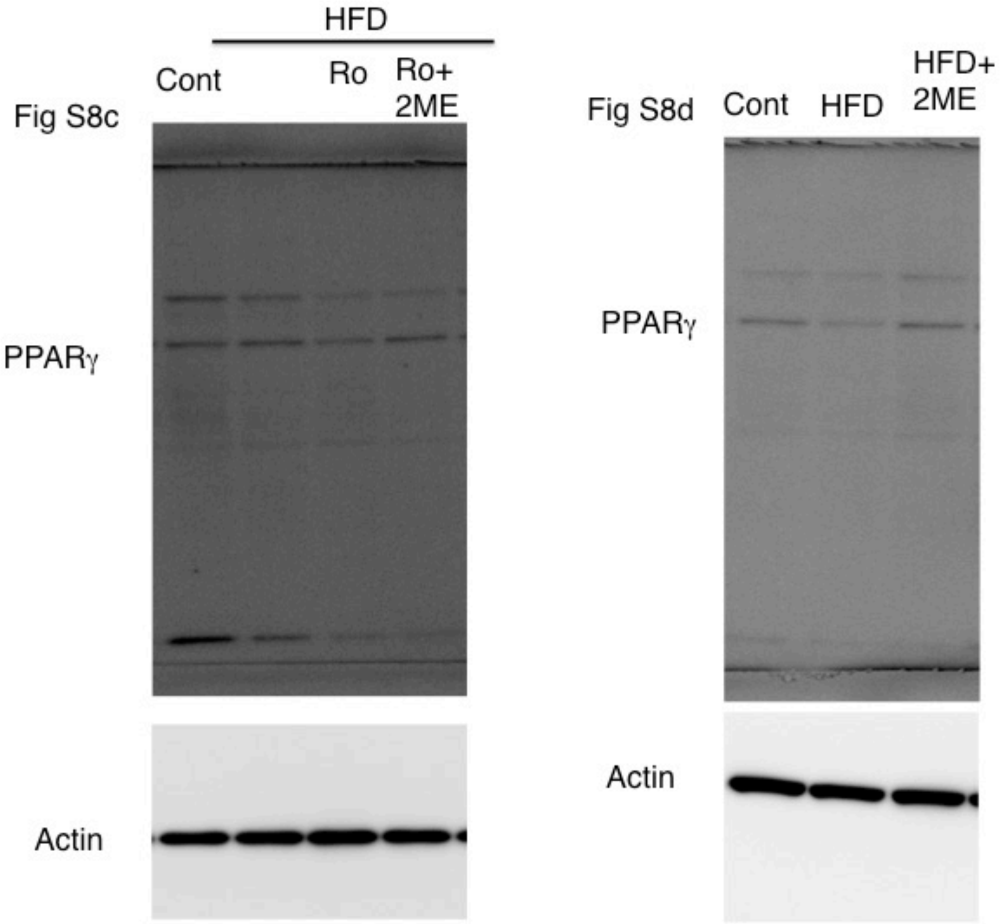

Fig. S21. Original blot for Fig S8 c and d

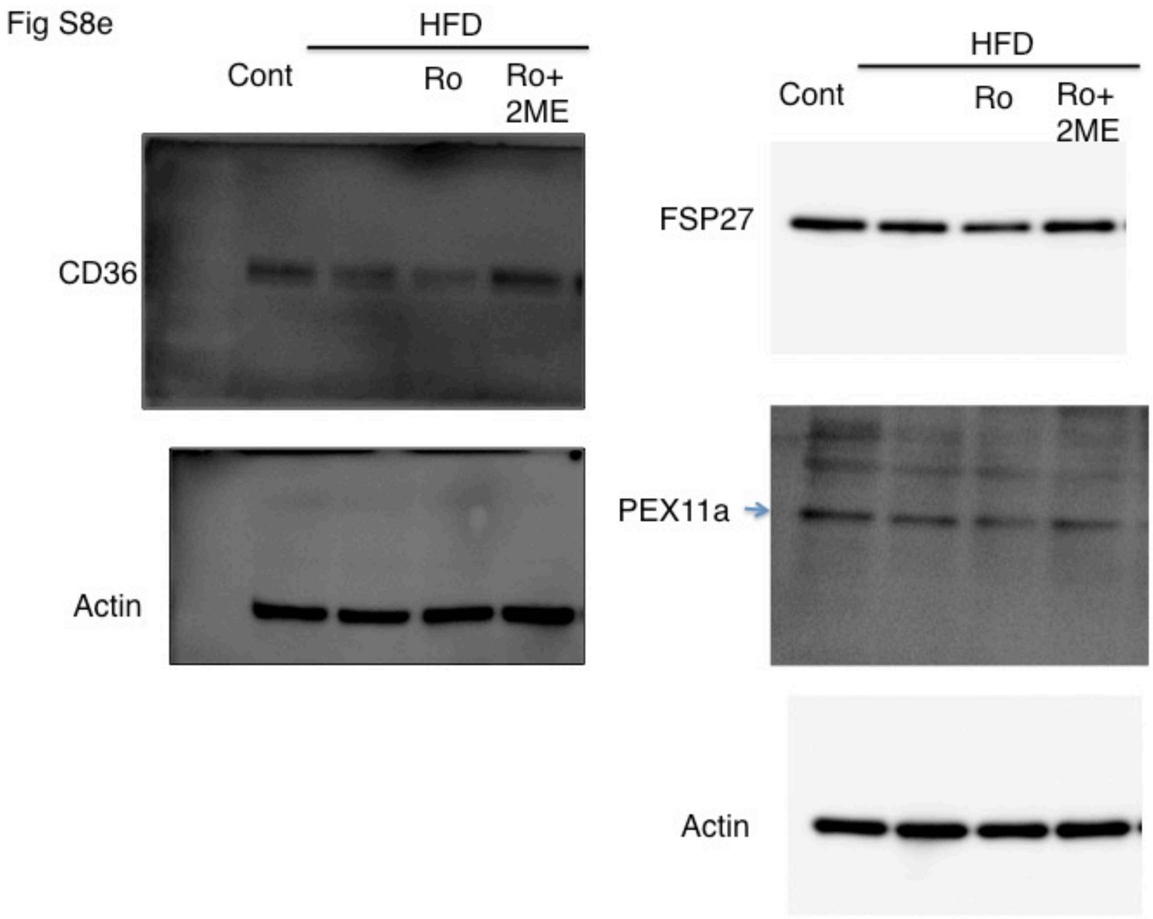

Fig. S21. Original blot for Fig S8e

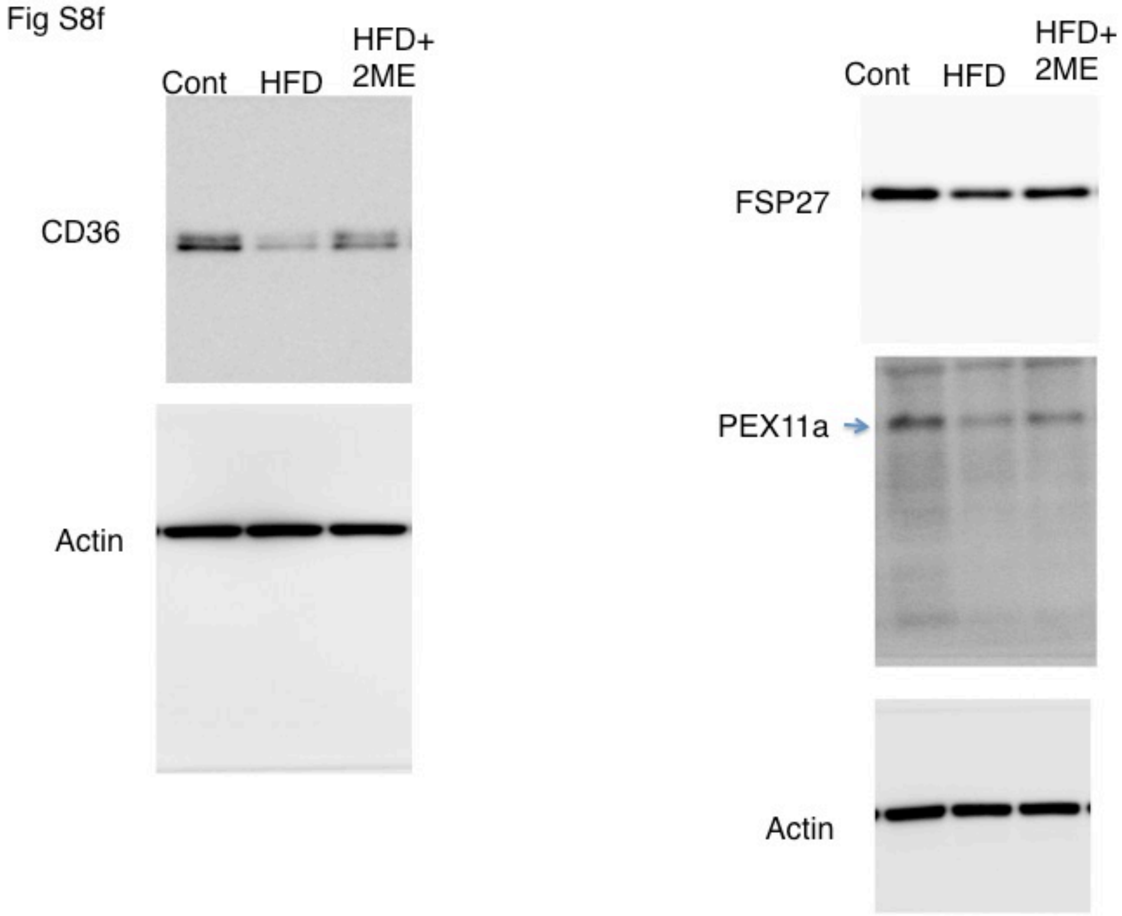

Fig. S22. Original blot for Fig S8f

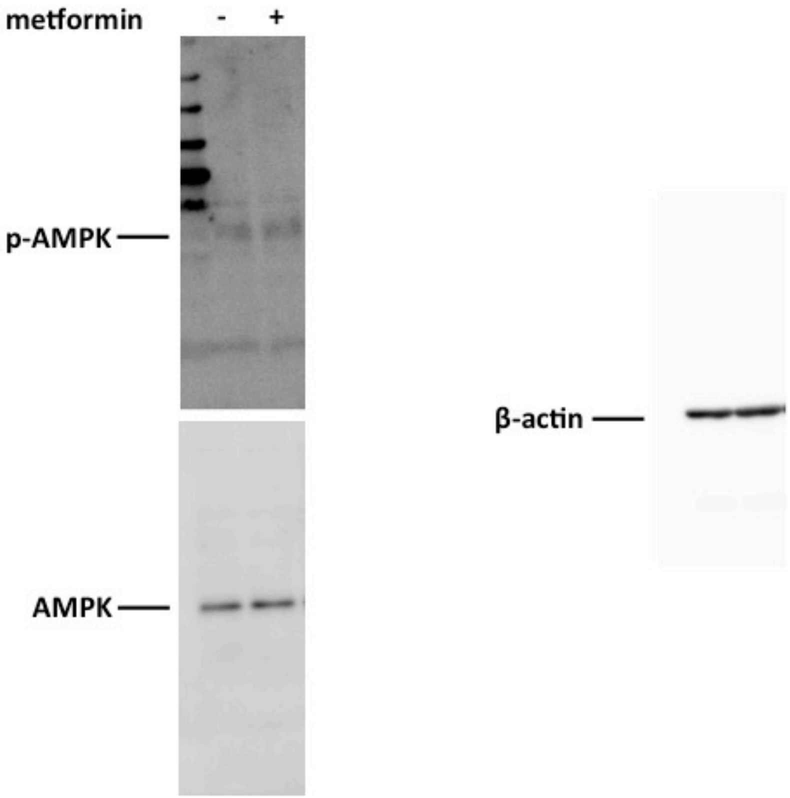

Fig. S23. Original blot for Fig S11
